# TIMP2 promotes AKI‐CKD transition by inducing tubular maladaptive repair and cell senescence via targeting Wnt/β‐catenin signalling

**DOI:** 10.1002/ctm2.70605

**Published:** 2026-01-28

**Authors:** Dongxue Xu, Haichuan Yu, Jingjing Pang, Xiaoyu Zhang, Jun Jiang, Yiming Li, Zhiyong Peng

**Affiliations:** ^1^ Department of Critical Care Medicine Zhongnan Hospital of Wuhan University Wuhan China; ^2^ Clinical Research Center of Hubei Critical Care Medicine Wuhan China; ^3^ Department of Critical Care Medicine University of Pittsburgh Pittsburgh Pennsylvania USA; ^4^ Intensive Care Unit of the second affiliated Hospital of Hainan Medical College Haikou China

**Keywords:** AKI, CKD, TIMP2

## Abstract

**Background:**

Acute kidney injury (AKI) frequently progresses to chronic kidney disease (CKD), but the underlying mechanisms of this transition remain unclear. While TIMP2 is a known biomarker for AKI, its direct pathogenic role in the AKI‐CKD transition has not been fully elucidated.

**Methods:**

TIMP2 expression was evaluated in multiple murine models, including unilateral ischemia‐reperfusion injury (UIR), unilateral ureteral obstruction (UUO), and cisplatin‐induced nephropathy. To investigate its function, we employed a tubule‐specific, inducible TIMP2 knockout mouse model (Ksp‐CreERT2; TIMP2fl/fl) and a tubular overexpression model.

**Results:**

TIMP2 was significantly upregulated during the AKI‐CKD transition across all tested models. Tubule‐specific deletion of TIMP2 markedly attenuated renal fibrosis, suppressed senescence‐associated secretory phenotypes (SASP), and promoted tubular repair. Conversely, TIMP2 overexpression exacerbated cellular senescence and fibrotic remodeling. Mechanistically, TIMP2 was found to bind to the Wnt co‐receptor LRP6, promoting its phosphorylation and subsequent β‐catenin signaling activation, a process independent of its canonical matrix metalloproteinase (MMP) inhibitory function.

**Conclusions:**

TIMP2 is a central mediator of maladaptive repair that links cell senescence and fibrotic reprogramming via the LRP6/β‐catenin pathway. These findings suggest that TIMP2 serves not only as a biomarker but also as a potential therapeutic target for mitigating the AKI‐CKD transition.

**Highlights:**

- TIMP2 is upregulated in injured renal tubules and promotes maladaptive repair and cell senescence.
- Genetic deletion of TIMP2 in tubular epithelial cells attenuates renal fibrosis and improves mitochondrial function.
- TIMP2 activates Wnt/β‐catenin signalling by binding to LRP6 via an MMP‐independent mechanism.

## Graphical abstract

1. TIMP2 is upregulated in injured renal tubules and promotes maladaptive repair and cell senescence.

2. Genetic deletion of TIMP2 in tubular epithelial cells attenuates renal fibrosis and improves mitochondrial function.

3. TIMP2 activates Wnt/β‐catenin signalling by binding to LRP6 via an MMP‐independent mechanism.

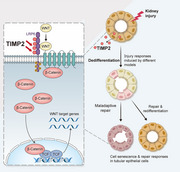

## 1 INTRODUCTION

Acute kidney injury (AKI) is increasingly prevalent and is associated with extended hospitalization and decreased survival.^1^, ^2^ Emerging evidence indicates that individuals with AKI face a heightened risk of progressing to chronic kidney disease (CKD) and, in some cases, end‐stage renal disease (ESRD).^3^, ^4^, ^5^ However, the mechanisms underlying the transition from AKI to CKD remain poorly understood. Renal tubular epithelial cells (RTECs) are particularly susceptible to acute injury.^6^, ^7^ Post‐injury repair mechanisms are classified as adaptive repair, restoring normal function after mild injuries, or maladaptive repair, leading to structural changes and declining kidney function. Maladaptive repair worsens kidney fibrosis and involves the release of pro‐fibrotic factors and increased extracellular matrix production.^8^, ^9^, ^10^, ^11^, ^12^ Mitochondrial dysfunction and disrupted homeostasis are characteristic features of kidney injury and maladaptive repair.^13^ Studies have shown that mitochondrial biogenesis is suppressed during maladaptive kidney repair, while its pharmacological activation enhances tubular regeneration and promotes renal recovery.^14^, ^15^ The mechanisms underlying this repair process are the subject of rigorous debate.^16^, ^17^

The tissue inhibitor of metalloproteinase‐2, TIMP2, is produced and secreted by RTECs and is notably elevated in the urine of early‐stage AKI, where it serves as a biomarker for tubular epithelial cell stress.^18^, ^19^, ^20^ Research from our laboratory has shown that during the early stages of sepsis‐induced AKI, TIMP2 regulates tubular pyroptosis.^21^ Furthermore, our previous study has demonstrated that TIMP2 promotes inflammatory responses in sepsis‐induced AKI.^22^, ^23^ In diabetic nephropathy, early elevation of TIMP2 contributes to podocyte apoptosis, thereby worsening renal injury.^24^ While the role of TIMP2 has been extensively studied in AKI, its long‐term effects and impact on fibrosis remain unclear. TIMP2 has been identified as a component of the ageing‐associated secretory phenotype (SASP),^25^ but the relationship between TIMP2 and cellular senescence and tubular repair has not yet been definitively established by existing literature.

The Wnt/β‐catenin signalling pathway is crucial for kidney repair after injury, known for its roles in development, proliferation and cell adhesion. Binding of Wnt ligands to Frizzled and LRP6 receptors suppresses the β‐catenin destruction complex, thereby stabilizing β‐catenin and promoting its translocation into the nucleus.^26^, ^27^ Recent studies suggest that the dysregulated expression of Wnt/β‐catenin is closely correlated with the advancement of cellular senescence and fibrosis.^28^, ^29^, ^30^

In this study, we delineated that TIMP2 promotes renal maladaptive repair and fibrosis via promoting tubular cell senescence, mechanistically through binding LRP6 to hyperactivate the canonical Wnt/β‐catenin pathway.

## 2 RESULTS

### 2.1 TIMP2 is induced in different models of AKI‐CKD

**FIGURE 1:**
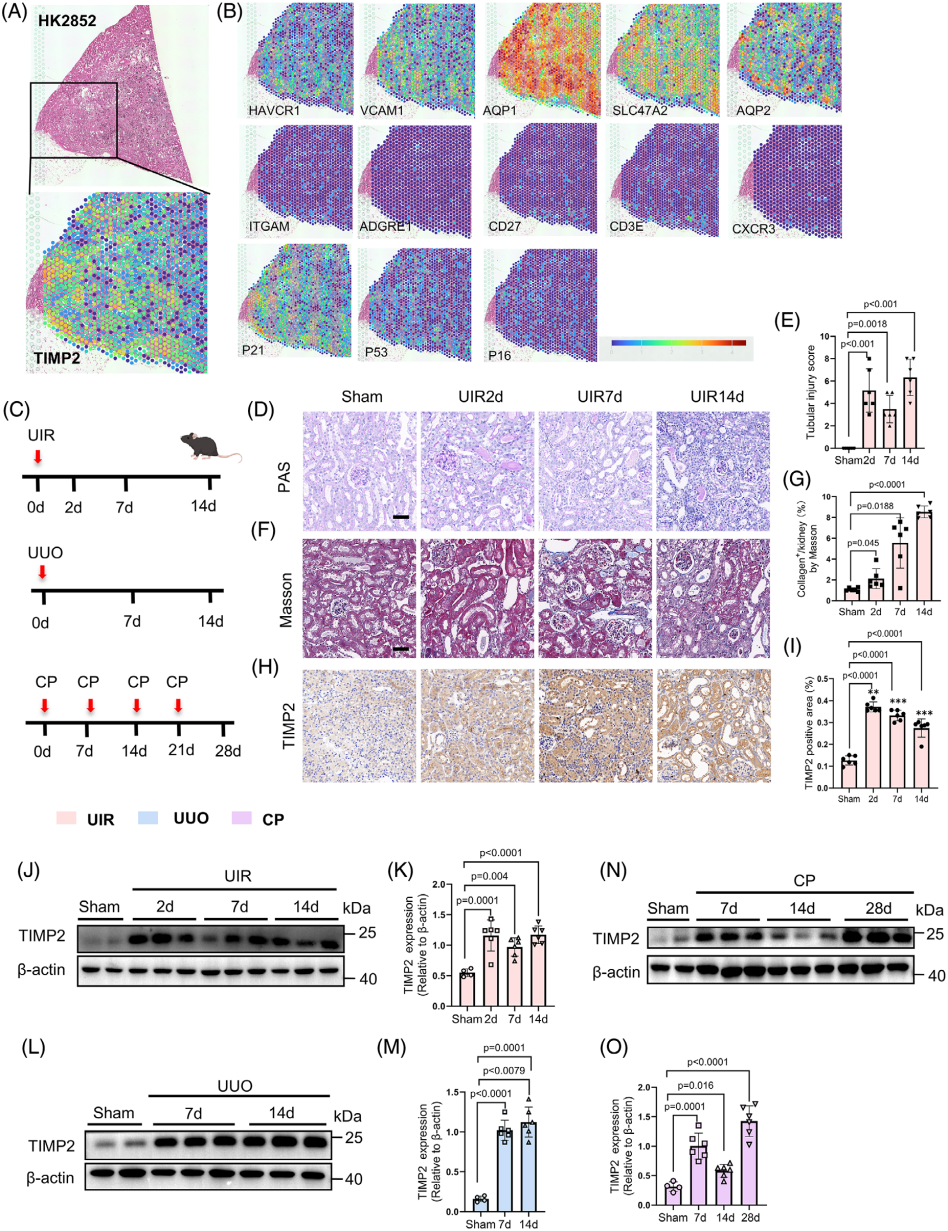
TIMP2 is induced in different models of AKI‐CKD. (A, B) Spatial location and marker gene expression using SP (Visium and CosMx), which include TIMP2, PT (SLC47A2, AQP1), injured PT (HACVR1, VCAM1), senescence‐associated markers in PT (CDKN1A, CDKN2A, p53), DT (AQP2), monocytes (CX3CR1, ITGAM), macrophages (ADGRE1), NK (CD27) and T cells (CD3E, CXCR3). The H&E section on the left of the figure showed tissue histology. Spatial location and marker gene expression analyzed from a publicly available human kidney spatial multi‐omics atlas (Sample ID: HK2852). Data were visualized using the Susztak Lab web tool (https://susztaklab.com/samui/. (C) Schematic diagram of the AKI‐CKD model establishment. Kidney tissues were harvested at specific time points: for the UIR model at days 2, 7 and 14; for the UUO model at days 7 and 14; and following cisplatin (8 mg/kg) injection at days 7, 14 and 28. (D) PAS staining of kidney tissues after UIR. The scale bar is 50 µm. (E) Quantification of injury tubule score (*n* = 6, data are mean ± SD, one‐way ANOVA test after Tukey's multiple‐comparison test). The overall ANOVA test revealed significant differences between groups (*F* = 23, *p* < .0001). (F) Masson staining of kidney tissues from three different CKD models. The scale bar is 50 µm. (G) Quantification of collagen fibre deposition in the kidney by Masson (*n* = 6, data are mean ± SD, one‐way ANOVA test after Tukey's multiple‐comparison test). The overall ANOVA test revealed significant differences between groups (*F* = 41.38, *p* = .0004). (H) Immunohistochemical staining demonstrates TIMP2 expression in the renal tubules. The scale bar is 50 µm. (I) Quantification of the percentage of TIMP2 in kidney tubules (*n* = 6, data are mean ± SD, one‐way ANOVA test after Tukey's multiple‐comparison test). The overall ANOVA test revealed significant differences between groups (F = 83.75, *p* = .0001). (J) WB analysis of TIMP2 protein expression levels at 2, 7 and 14 days following UIR. (K) Quantitative analysis of TIMP2 protein expression levels by Western blot on day 2, 7 and 14 post‐UIR (*n* = 6). (L) WB analysis of TIMP2 protein expression levels at 7 days and 14 days following UUO. Red signal indicates TIMP2. (M) Co‐staining with LTL (green) shows TIMP2 localization in proximal tubules. (N) Co‐staining with KIM‐1 (green) confirms expression in injured proximal tubules. (O) Co‐staining with Cdh16 (green) reveals robust TIMP2 expression in the distal nephron. (P) Co‐staining with AQP2 (green) further confirms TIMP2 expression in the collecting ducts. Scale bar = 20 µm. The widespread co‐localization with Cdh16 and AQP2 validates the use of the Ksp‐Cre driver. (Q) Quantitative analysis of TIMP2 protein expression levels by Western blot on day 7 and 14 post‐UUO (*n* = 6, data are mean ± SD, one‐way ANOVA test after Tukey's multiple‐comparison test). The overall ANOVA test revealed significant differences between groups (*F* = 12.44, *p* < .0001). (R) WB analysis of TIMP2 protein expression levels at 7, 14 and 28 days post‐CP treatment. (S) Quantitative analysis of TIMP2 protein expression levels by Western blot on day 7, 14 days and 28 days post‐CP (*n* = 6, data are mean ± SD, one‐way ANOVA test after Tukey's multiple‐comparison test). The overall ANOVA test revealed significant differences between groups (*F* = 33.54, *p* < .0001). Group sizes refer to biological replicates.

To assess TIMP2 expression in the human kidney, we utilized a publicly available, high‐resolution spatial multi‐omics atlas of human renal tissue, which enables detailed mapping of cellular distribution and gene expression under physiological and pathological conditions.^31^ As shown in Figure 1A,B, cell populations were delineated on an H&E‐stained kidney biopsy obtained from a patient with kidney disease (HK2852). We observed that TIMP2 expression was highly enriched within the injured tubular epithelial clusters (identified by the co‐expression of SLC47A2/AQP1 with injury markers HACVR1/VCAM1). This expression pattern was distinct from various immune populations, including monocytes (CX3CR1, ITGAM), macrophages (ADGRE1), T cells (CD3E, CXCR3) and NK cells (CD27). Notably, senescence‐associated markers (CDKN1A, CDKN2A, p53) were also spatially enriched in these injured tubular clusters, with CDKN1A displaying a distribution pattern closely paralleling that of TIMP2. Collectively, these findings indicate that TIMP2 expression is significantly upregulated within the injured tubular microenvironment.

To investigate whether TIMP2 expression correlates with AKI progression in vivo, we assessed the TIMP2 expression in established murine models of AKI–CKD^32^: unilateral ischemia‐reperfusion (UIR), unilateral ureteral obstruction (UUO) and cisplatin‐induced nephropathy (CP) (Figure 1C). Histopathological assessment using PAS staining demonstrated significant tubular atrophy and dilation (Figure 1D,E; Figure S1A,B,G–J), while Masson's trichrome staining confirmed progressive tubulointerstitial fibrosis (Figure 1F,G; Figure S1C,D,H–K) across all three models. Immunohistochemical (IHC) staining revealed distinct TIMP2 protein accumulation specifically localized to renal tubules in injured kidneys compared with Sham controls (Figure 1H,I; Figure S1E,F,I–L). To strictly validate the cellular source of TIMP2 protein, we examined TIMP2 localization using segmental markers. Immunofluorescence analysis revealed that TIMP2 protein was upregulated in the tubular compartment following UIR. We detected TIMP2 signal in LTL‐positive proximal tubules and co‐localized with the injury marker KIM‐1 (Figure S1M,N). Notably, however, the most prominent TIMP2 expression was observed in Cdh16‐positive distal tubules and AQP2‐positive collecting ducts (Figure S1O,P). Given that the Ksp‐Cre driver targets the Cdh16^+^ lineage, this specific enrichment pattern supports the physiological relevance of our genetic model in dissecting the role of tubular TIMP2.

Furthermore, Western blot analysis confirmed a time‐dependent and significant induction of total TIMP2 protein levels following injury (Figure 1J–O; Figure S1Q–S). Consistent with protein data, Timp2 mRNA upregulation was observed across all models (Figure S1Q–S). These findings suggest that TIMP2 upregulation, particularly in injured tubules, is a prominent feature of maladaptive repair following AKI.

### 2.2 Genetic deletion of TIMP2 reduces maladaptive repair after AKI

**FIGURE 2:**
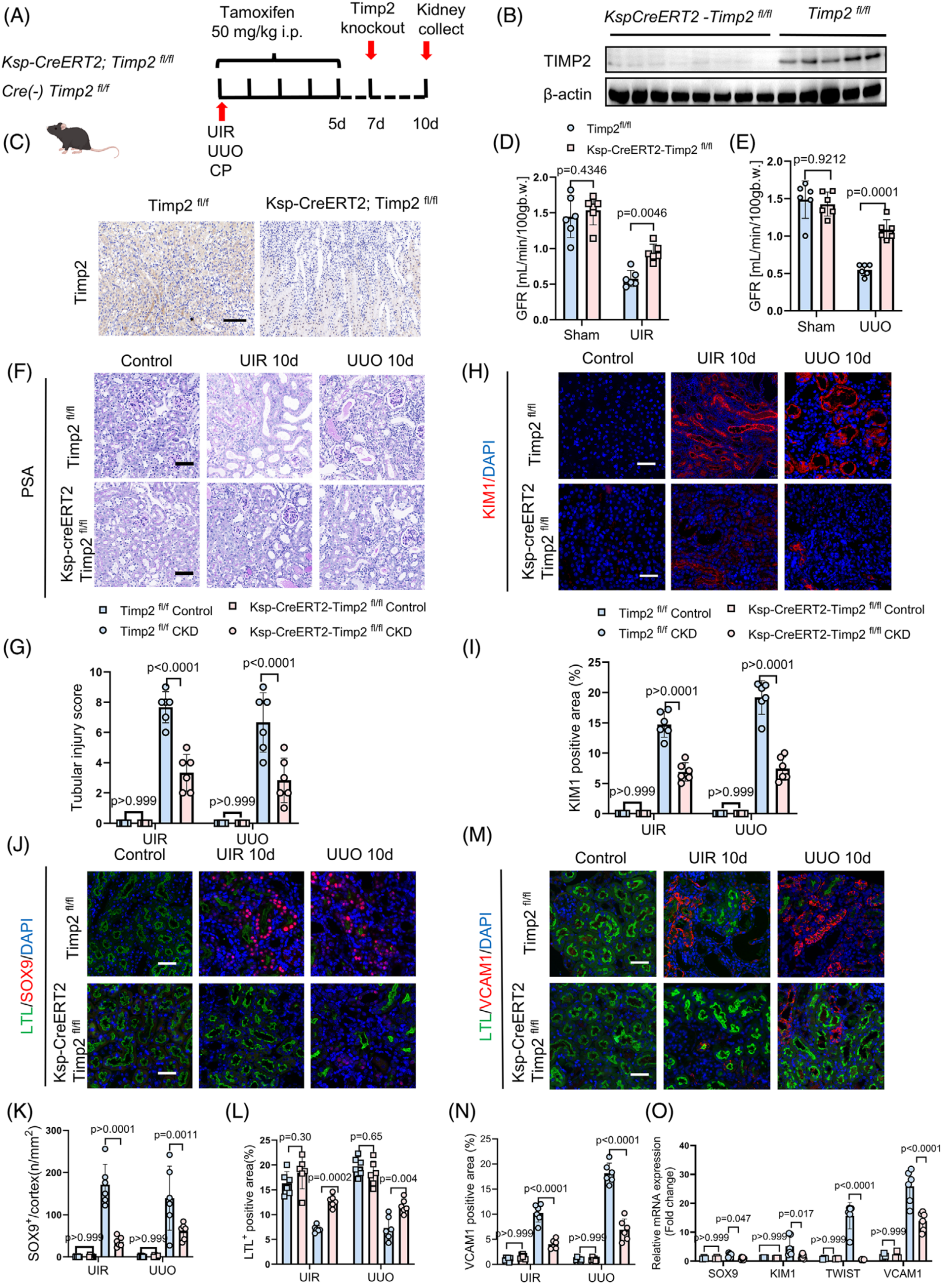
Loss of TIMP2 promotes maladaptive repair during tubular recovery post‐injury. (A) Illustration of TIMP2 gene knockout in *Ksp‐creERT2; TIMP2^flox/flox^* mice: animals received 50 mg/kg tamoxifen by daily intraperitoneal injection for five consecutive days, with tissue harvested on day 10. (B) Western blot of TIMP2 protein levels in TIMP2^flox/flox^ and Timp2 knockout (*Ksp‐creERT2; TIMP2^flox/flox^*) mice after tamoxifen injection. (C) Immunohistochemical staining demonstrates TIMP2 expression in the renal tubules of *Ksp‐creERT2; TIMP2^flox/flox^* and *TIMP2^flox/flox^* mice. The scale bar is 50 µm. (D) Dynamic change in glomerular filtration rate after UIR in *Ksp‐creERT2; TIMP2^flox/flox^* and *TIMP2^flox/flox^* mice (*n* = 3, data are mean ± SD, two‐way ANOVA test after Tukey's multiple‐comparison test). For the UIR model: overall ANOVA revealed a significant Interaction between genotype (F (1, 20) = 16.79, *p* < .0001), as well as significant main effects for treatment (F (1, 20) = 118.6, *p* < .0001) and Interaction (F (1, 20) = 8.52, *p* < .0001). (E) Dynamic change in glomerular filtration rate after UUO in Ksp‐creERT2; TIMP2^flox/flox^ and TIMP2^flox/flox^ mice, *n* = 3, data are mean ± SD, two‐way ANOVA test after Tukey's multiple‐comparison test. UIR: interaction F (1, 20) = 87.77, *p* < .0001; treatment *p* < .0001; genotype *p* < .0001. UUO: interaction F (1, 20) = 48.75, *p* < .0001; treatment *p* < .0001; genotype *p* = 0.0003. (F, G) PAS staining reveals attenuated tubular injury and interstitial damage in Timp2 knockout mice across multiple CKD models compared with control, *n* = 6, data are mean ± SD, two‐way ANOVA test. UIR: interaction F (1, 20) = 44.4, *p* < .0001; treatment *p* < .0001; genotype *p* < .0001. UUO: interaction F (1, 20) = 6.15, *p* < .022; treatment *p* < .0001; genotype *p* < .023. (H) Immunofluorescence staining showing the expression of the kidney injury molecule‐1 (KIM‐1) in *TIMP2^flox/flox^* and Timp2 knockout (*Ksp‐creERT2; TIMP2^flox/flox^*) mice within the UIR model. (I) Quantitative analysis of KIM1+ area/cortex (%). *n* = 6. Data are mean ± SD, one‐way ANOVA test. UIR: interaction F (1, 20) = 87.77, *p* < .0001; treatment *p* < .0001; genotype *p* < .0001. UUO: interaction F (1, 20) = 135.2, *p* < .0001; treatment *p* < .0001; genotype *p* < .0001. (J) Immunofluorescence staining displaying the dual labelling of lotus tetragonolobus lectin (LTL), which marks intact proximal tubule brush borders, and SOX9, a transcription factor involved in development and cell differentiation. The scale bar is 50 µm. (K) Quantitative analysis of SOX9^+^area/cortex (%). *n* = 6, data are mean ± SD, two‐way ANOVA test after Tukey's multiple‐comparison test. UIR: interaction F(1, 20) = 44.43, *p* < .0001; treatment *p* < .0001; genotype *p* < .0001. UUO: interaction F(1, 20) = 6.15, *p* = .022; treatment *p* < .0001; genotype *p* = .023. **(L)** Quantitative analysis of LTL^+^ area/cortex (%). *n* = 6. Data are mean ± SD, two‐way ANOVA test after Tukey's multiple‐comparison test. UIR: interaction F (1, 20) = 44.43, *p* < .0001; treatment *p* < .0001; genotype *p* < .0001. UUO: interaction F (1, 20) = 6.15, *p* = .022; treatment *p* < .0001; genotype *p* = .023. (M) Immunofluorescence staining of vascular cell adhesion molecule 1 (VCAM1) across TIMP2^flox/flox^ and Timp2 knockout mice subjected to Sham, UIR and UUO treatments. The scale bar is 50 µm. (N) Quantitative analysis of VCAM1+ area/cortex (%). *n* = 6. Data are mean ± SD, two‐way ANOVA test after Tukey's multiple‐comparison test. UIR: interaction F (1, 20) = 66.86, *p* < .0001; treatment *p* < .0001; genotype *p* < .0001. UUO: interaction F (1, 20) = 101.5, *p* < .0001; treatment *p* < .0001; genotype *p* < .0001. (O) qPCR analysis of mRNA expression levels of Sox9, Havcr (Kidney injury molecule‐1, KIM‐1), Twist and Vcam1 in the UIR models of *TIMP2^flox/flox^* and Timp2 knockout mice, *n* = 3–6. Data are mean ± SD, two‐way ANOVA test after Tukey's multiple‐comparison test. SOX9: interaction F (1, 14) = 5.75, *p* = .031. KIM1: interaction F (1, 14) = 10.51, *p* = .006. TWIST: interaction F (1, 14) = 50.85, *p* < .0001. VCAM1: interaction F (1, 14) = 22.13, *p* < .001. Group sizes refer to biological replicates.

To investigate the role of TIMP2 in the tubular repair dynamics process following injury, we generated inducible *Ksp‐CreERT2; Timp2^fl/fl^* mice to allow for controlled deletion of TIMP2 in renal tubular epithelial cells after the initiation of injury. As depicted in the experimental schema (Figure 2A), mice were subjected to UIR, UUO, or CP injury, followed by tamoxifen administration from Days 1–5 post‐injury. This strategy facilitates a detailed examination of TIMP2's function during the critical window of kidney repair. Efficient TIMP2 knockout was confirmed by Western blot, qPCR and IHC at Day 7 (Figure 2B,C; Figure S2A,B). Renal function was assessed at the endpoint (Day 10). The transcutaneous real‐time measurement of glomerular filtration rate (GFR) was significantly impaired in Timp2^fl/fl^ mice at day 14 across all three models, whereas TIMP2‐deficient mice (*Ksp‐CreERT2; Timp2^fl/fl^*) exhibited preserved renal function, indicating a protective effect of TIMP2 deletion (Figure 2D,E; Figure S2C). Histological assessment via PAS staining showed that TIMP2 deletion significantly attenuated the tubular injury score across all three models compared with littermate controls (Figure 2F,G). Correspondingly, the expression levels of kidney injury molecule‐1 (KIM‐1), a well‐recognized marker of tubular injury, were attenuated in TIMP2‐deficient kidneys following UIR, UUO and CP (Figure 2H,I; Figure S2D–F,G,H). Notably, the number of dedifferentiated Sox9^+^ proximal tubular cells—a tubular hallmark of failed repair—was markedly reduced in the kidney of *Ksp‐creERT2‐Timp2^fl/fl^* mice (Figure 2J,K; Figure S2I,J). Conversely, the loss of LTL^+^ brush borders, a marker of differentiated proximal tubules, was significantly mitigated in TIMP2‐deficient kidneys, suggesting enhanced preservation of the epithelial phenotype (Figure 2J–L; Figure S2K–M). The maladaptive repair marker VCAM1 observed in *Timp2^fl/fl^* kidneys was significantly blunted in the knockout group (Figure 2M,N; Figure S2J–L). Additionally, RT‐qPCR analysis further confirmed that the upregulation of cell dedifferentiation and fibrotic markers—including α‐Sma, Vim, Havcr1, Sox9, Twist and Vcam1—was significantly downregulated in *Ksp‐CreERT2; Timp2^fl/fl^* kidneys after AKI (Figure 2O). Overall, these findings suggest that TIMP2 contributes to maladaptive repair during AKI‐CKD.

### 2.3 Loss of TIMP2 alleviates fibrosis and injury in CKD models

**FIGURE 3:**
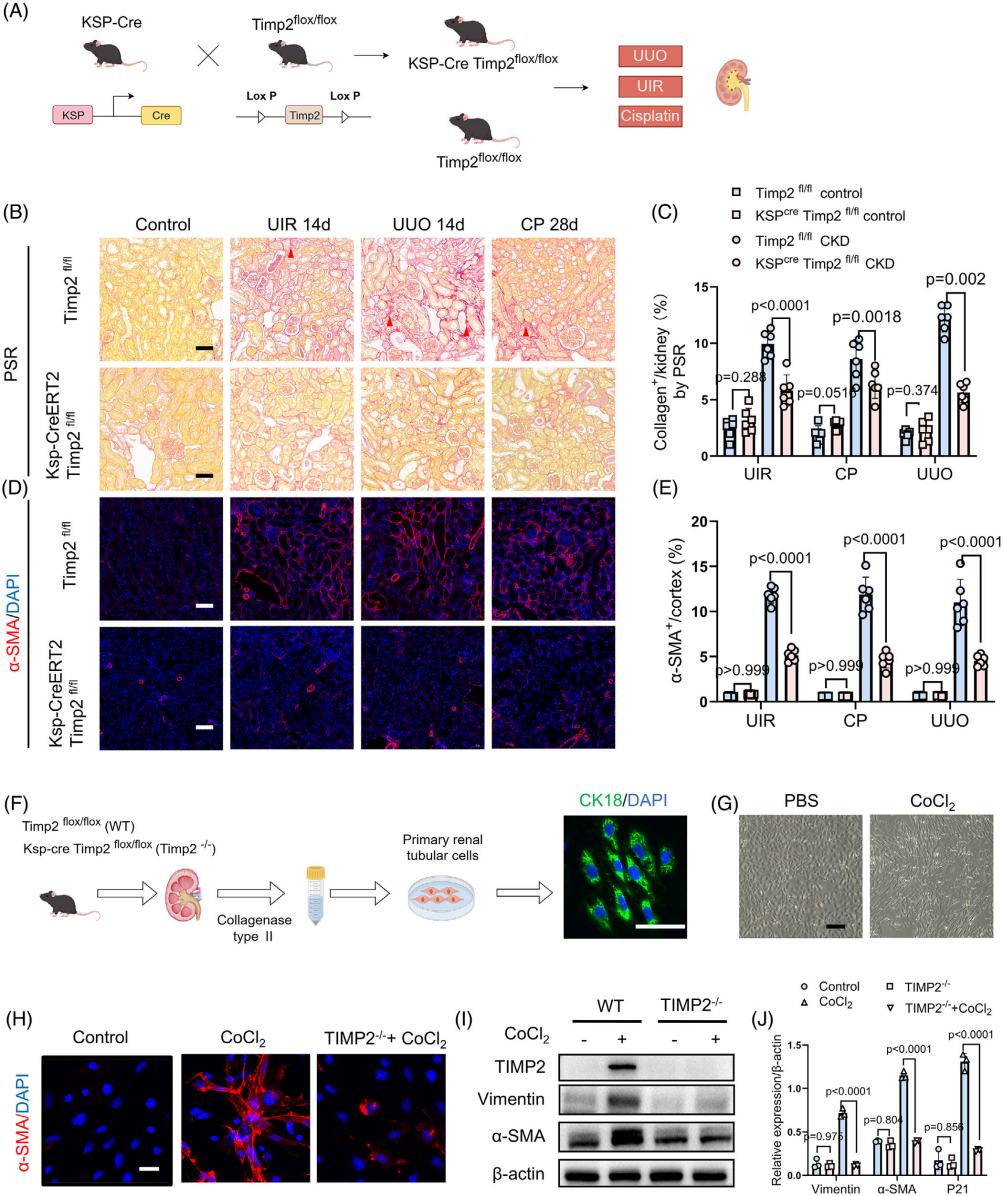
Genetic deletion of TIMP2 reduces fibrosis and injury in CKD Models. (A) Illustration of TIMP2 gene knockout in *Ksp‐Cre; TIMP2^flox/flox^* mice. CKD models were induced by UIR and UUO (tissues collected on day 14) and by CP nephropathy (samples on day 28). (B) Picrosirius red (PSR) staining illustrates the distribution of collagen fibres (shown in red) in kidney tissues from mice in Sham, UIR, CP and UUO groups. The scale bar represents 50 µm. (C) Quantification of collagen fibre deposition in the kidney by PSR. *n* = 6, data are mean ± SD, two‐way ANOVA test. UIR: interaction F (1, 20) = 31.42, *p* < .0001. CP: interaction F (1, 20) = 7.85, *p* = .011. UUO: interaction F (1, 20) = 93.56, *p* < .0001. (D) Immunofluorescent staining for alpha‐smooth muscle actin (α‐SMA) in Sham, UIR, CP and UUO groups. Scale bar = 50 µm. (E) Quantitative statistics are displayed, highlighting the differences in α‐SMA expression across the experimental conditions. *n* = 6, data are mean ± SD, two‐way ANOVA test. UIR: interaction F (1, 20) = 142.5, *p* < .0001; treatment *p* < .0001; genotype *p* < .0001. CP: interaction F (1, 20) = 68.45, *p* < .0001; treatment *p* < .0001; genotype *p* < .0001. UUO: interaction F (1, 20) = 55.62, *p* < .0001; treatment *p* < .0001; genotype *p* < .0001. (F) Schematic of primary mouse renal tubular cell isolation and identified/verified using the renal tubular marker cytokeratin 18 (CK18). Scale bar = 100 µm. (G) Microscope bright‐field images of primary renal tubular cells with or without CoCl_2_ (200 µM). stimulation. CoCl_2_ treatment was used to establish a hypoxia‐reoxygenation model. (H) Immunofluorescence staining demonstrating the expression of α‐SMA in proximal renal tubular cells (PRTCs). (I) Western blot analysis of fibronectin (FN) and α‐SMA protein expression in WT and TIMP2 TIMP2^−/−^ PRTCs stimulated with CoCl_2_(200 µM). (J) Quantitative analysis of TIMP2, Vimentin and α‐SMA protein expression levels by Western blot (*n* = 3, data are mean ± SD, two‐way ANOVA test). Vimentin: interaction F (1, 8) = 100.9, *p* < .0001. α‐SMA: interaction F (1, 8) = 328.7, *p* < .0001. P21: interaction F (1, 8) = 113.7, *p* < .0001. Specific comparisons indicate that the hypoxia‐induced upregulation of fibrosis and senescence markers was significantly blunted in TIMP2‐deficient PRTCs (*p* < .001 vs. WT‐CoCl2 group). Group sizes refer to biological replicates. Experiments were repeated in three biological replicates using primary cells isolated from different mice.

To further validate TIMP2's role in CKD progression, we employed constitutive tubule‐specific TIMP2 knockout mice (*KspCre; TIMP2^fl/fl^*) and subjected them to three AKI‐CKD models: UIR, UUO and cisplatin nephropathy (CP) (Figure 3A; Figure S3A). Masson's trichrome and Picrosirius red (PSR) staining revealed reduced collagen deposition and attenuated tubular injury in Timp2 knockout mice across CKD models (Figure 3B,C; Figure S3B,C). Additionally, immunofluorescent staining showed smooth muscle actin (α‐SMA) and fibrosis markers, including fibronectin 1(FN1), were elevated in the *Timp2^fl/fl^*—CKD group compared with the Timp2 knockout CKD mice (Figure 3D,E; Figure S3D,E), consistent with protein quantification by Western blot (Figure S3F,G).

We next isolated primary renal tubular epithelial cells (PRTCs) from *Timp2^fl/f^* ^l^ (WT) and *KspCre‐Timp2^fl/fl^* (Timp2^−/−^) mice. Cells were confirmed by immunofluorescent staining for cytokeratin 18 (CK18), a specific marker of renal tubules (Figure 3F). PRTCs were subjected to chemical hypoxia using cobalt chloride (CoCl_2_) with 200 µM CoCl_2_ for 12 h to mimic ischemic conditions, followed by 12 h of reoxygenation in complete medium under normoxic conditions (21% O_2_)^33^ (Figure 3G). In WT PRTCs, CoCl_2_ treatment resulted in elevated levels of fibrosis markers, specifically α‐SMA and Vimentin. In contrast, TIMP2‐deficient PRTCs maintained a more epithelial morphology and exhibited significantly lower induction of α‐SMA and Vimentin protein levels upon hypoxic stress (Figure 3H–J). Collectively, these findings indicate that Timp2 deletion in both mice and tubular epithelial cells exerts a protective effect by mitigating tubular injury and interstitial fibrosis, facilitating renal repair, and thereby confers protection against the progression from AKI to CKD.

### 2.4 TIMP2 deletion reduces cell senescence and mitochondrial dysfunction

**FIGURE 4:**
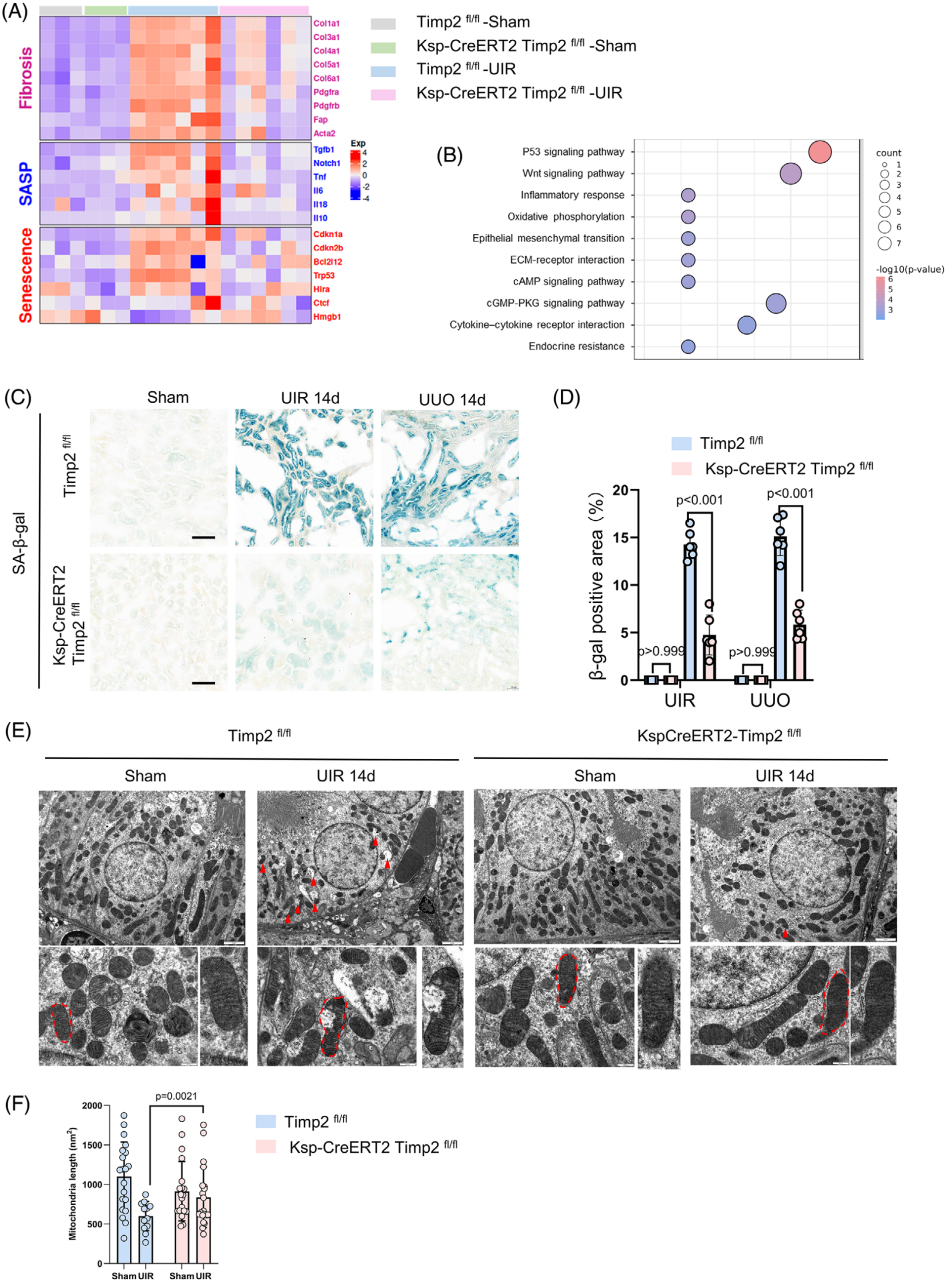
Reduced cell senescence and mitochondrial dysfunction in absence of TIMP2. (A) Heatmap depicting the expression changes of genes related to fibrosis, senescence‐associated secretory phenotype (SASP) and senescence in Timp2^fl/fl^ and Timp2 knockout mice under the unilateral ischemia‐reperfusion (UIR) model. Red indicates upregulated expression, while blue indicates downregulated expression. (B) KEGG analysis showing biological processes involved by upregulated genes in the kidney from *Timp2^fl/fl^* –UIR group versus *Ksp‐CreERT2 Timp2 ^fl/fl^* –UIR. (C) SA‐β‐gal staining marking senescent cells in the kidneys of *Timp2 ^fl/fl^* and *KSPCre‐Timp2^fl/fl^* mice in UIR and UUO models compared with the sham group. Scale bar is 50 µm. (D) Quantitative analysis of SA‐β‐gal staining. *n* = 6. Data are mean ± SD. Two‐way ANOVAs followed by Tukey's post hoc test. UIR: interaction F (1, 20) = 227.0, *p* < .0001; treatment *p* < .0001; genotype *p* < .0001. UUO: interaction F (1, 20) = 175.7, *p* < .0001; treatment *p* < .0001; genotype *p* < .0001. (E) Representative transmission electron microscopy (TEM) images of tissue sections. Red arrows: Indicating mitochondrial damage. Red dashed boxes: Areas shown in magnified insets. (F, G) Quantitative analysis of mitochondrial morphology using ImageJ: Measured parameters: Size (diameter) and cross‐sectional area. Sampling method: ≈20 images randomly selected per sample. Data are mean ± SD. Two‐way ANOVAs followed by Tukey's post hoc test. Mitochondria length: interaction F (1, 86) = 14.35, *p* < .001; treatment F (1, 86) = 153.2, *p* < .0001; genotype F (1, 86) = 18.56, *p* < .0001.

TIMP2 has been recognized as a constituent of the senescence‐associated secretory phenotype (SASP),^25^ which plays a crucial role in tubular repair.^34^ To assess whether TIMP2 deficiency attenuates tubular cell senescence, we performed transcriptomic analysis of renal cortex RNA from *Timp2^fl/fl^* and *Ksp‐Cre; Timp2^fl/fl^* mice in the UIR‐induced AKI‐CKD model. Heatmap analysis revealed a significant upregulation of fibrosis markers (Col1a1, Col3a1, Col4a1, Col5a1, Col6a1), senescence markers (Cdkn1a, Cdkn2b, Bcal2l12, Trp53, Hmgb1) and SASP genes (Tgfb1, Il6, Il18, Il10) in the UIR model of *Timp2^fl/fl^* mice compared with the Sham group, while Timp2 knockout mice exhibited decreased expression pathways linked to cellular senescence were enriched in *Timp2^fl/fl^* but significantly downregulated in the TIMP2 knockout group especially of ageing‐related genes (Figure 4A). Gene Ontology analysis further revealed that enrichment of pathways linked to cellular senescence and in *Timp2^fl/fl^* mice, which were significantly downregulated in the TIMP2 knockout group, underscoring the role of TIMP2 in senescence‐associated processes (Figure 4B). SA‐β‐gal staining indicated reduced positive area in Timp2 knockout mice in both UIR and UUO models (Figure 4C,D). Western blot analysis revealed that P21 and γH2AX expression were significantly attenuated after UIR and UUO, but nearly abolished in TIMP2 knockdown mice (Figure S4A–D). These results were confirmed by qPCR data (Figure S4E). Given the established link between mitochondrial dysfunction and cellular senescence, we next examined mitochondrial integrity. Interestingly, we observed that mitochondrial ultrastructure in renal tissue morphology was markedly improved following TIMP2 deletion. In TIMP2^fl/fl^‐UIR mice, mitochondria appeared swollen with disrupted and fragmented cristae. In contrast, mitochondria in the TIMP2‐deficient group showed reduced damage, maintaining a more normal round or rod‐like shape with relatively well‐organized cristae (Figure 4E,F).

To investigate whether senescent cells are pivotal in post‐injury renal repair and fibrosis, we tested the senolytic agent,^32^, ^35^ ABT‐263, in the UIR mouse model of kidney fibrosis (Figure S5A). Histological analysis revealed reduced tissue scarring and collagen accumulation in ABT‐263‐treated mice (Figure S5B–E). SA‐β‐gal staining indicated fewer positive areas in ABT‐263‐treated mice in the UIR model (Figure S5F,G). Additionally, macrophage infiltration was significantly lower in treated mice, indicating reduced renal inflammation (Figure S5H,I). Immunofluorescent staining revealed that α‐SMA and VCAM1 were increased in WT mice but lower in mice treated with ABT‐263 (Figure S5J–N). Western blot analysis confirmed a significant reduction in fibrosis and damage markers FN, α‐SMA, KIM‐1 and γ‐H2AX, suggesting that ABT‐263 alleviated renal damage and interstitial fibrosis (Figure S5O,P). These findings provide further evidence that sustained tubular cell senescence contributes to maladaptive repair and the progression of CKD.

### 2.5 TIMP2 regulates cellular senescence and mitochondrial repair through MMP‐independent mechanisms

**FIGURE 5:**
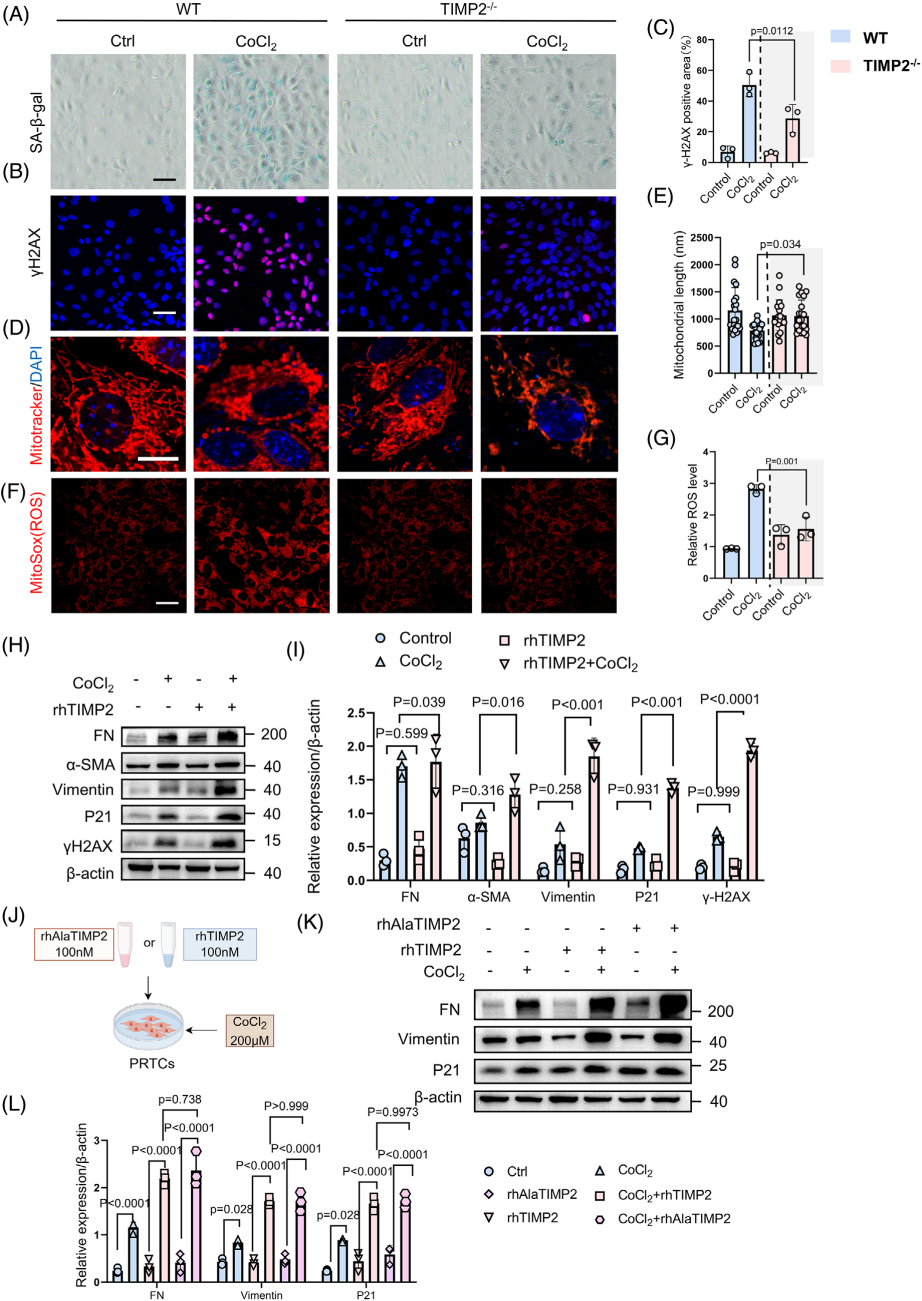
TIMP2 regulates cellular senescence and maladaptive repair through mechanisms that are independent of MMPs. (A) SA‐β‐gal activity in PRTCs from WT and Timp2^−/−^ mice, with or without CoCl_2_ stimulation. Scale bar is 20 µm. (B) Immunofluorescence staining showing the expression of γ‐H2AX in PRTCs. Scale bar is 20 µm. (C) Quantification of γ‐H2AX fluorescence. *n* = 3. Data are mean ± SD. Statistical significance was assessed by two‐way ANOVA followed by Tukey's post hoc test. γ‐H2AX: interaction F (1, 8) = 10.96, *p* = .011; treatment F (1, 8) = 107.0, *p* < .0001; genotype F (1, 8) = 11.66, *p* = .009. Group sizes refer to biological replicates. (D) Confocal microscopy analysis of mitochondrial morphology in PRTCs isolated from WT and Timp2^−/−^ mice. Cells were treated with or without 200 µM CoCl_2_ for 24 h to induce hypoxic stress. Scale bar is 100 µm. (E) Quantitative analysis of mitochondrial length using ImageJ: Measured parameters. Sampling method: ≈20 images randomly selected per sample. Scale bars: 10 µm. Data are mean ± SD. Statistical significance was assessed by two‐way ANOVA followed by Tukey's post hoc test. Mitochondrial length: interaction F (1, 76) = 12.35, *p* < .001; treatment F (1, 76) = 15.82, *p* < .001; genotype F (1, 76) = 9.47, *p* = .003. Specific comparisons indicate that mitochondrial length was significantly preserved in TIMP2‐deficient cells under hypoxic stress. (F) MitoSox staining in WT, TIMP2^−/−^ ‐cells treated with CoCl_2_. Scale bars: 20 µm. (G) Quantification of MitoSox fluorescence. *n* = 3. Group sizes refer to biological replicates. Data are mean ± SD. Statistical significance was assessed by two‐way ANOVA followed by Tukey's post hoc test. MitoSOX fluorescence: interaction F (1, 8) = 48.06, *p* = .0001; treatment F (1, 8) = 74.07, *p* < .0001; genotype F (1, 8) = 7.72, *p* = .024. Specific comparisons indicate that the hypoxia‐induced accumulation of mitochondrial ROS was significantly blunted in TIMP2‐deficient cells. (H, I) Quantitative statistics of FN, α‐SMA, Vimentin, P21 and γ‐H2AX protein levels in the PRTCs after treatment with CoCl_2_ and either recombinant human TIMP2 (rhTIMP2). *n* = 3. Data are mean ± SD. Group sizes refer to biological replicates. Statistical significance was assessed by two‐way ANOVA followed by Tukey's post hoc test. FN: interaction F (1, 8) = 6.27, *p* = .037. α‐SMA: interaction F (1, 8) = 10.37, *p* = .012. Vimentin: interaction F (1, 8) = 108.6, *p* < .0001. P21: interaction F (1, 8) = 98.54, *p* < .0001. γ‐H2AX: interaction F (1, 8) = 125.4, *p* < .0001. Specific comparisons indicate that rhTIMP2 treatment significantly exacerbated the expression of senescence and fibrosis markers under hypoxic conditions. (J) Schematic representation of the experimental design: PRTCs after treatment with CoCl_2_ (200 µM) and either recombinant human TIMP2 (rhTIMP2) or its mutant rhAlaTIMP2 (100 nM). rhTIMP2 refers to recombinant human TIMP2 protein, while rhAlaTIMP2 is a mutant variant of human TIMP2 that lacks MMP inhibition functionality. (K, L) Quantitative statistics of FN, Vimentin and P21 protein levels in the HK‐2 cell line after treatment with CoCl_2_ (200 µM) and either recombinant human TIMP2 (rhTIMP2) or its mutant rhAlaTIMP2 (100 nM). *n* = 3. Data are mean ± SD. Statistical significance was assessed by two‐way ANOVA followed by Tukey's post hoc test. FN: interaction F (2, 12) = 105.4, *p* < .0001. Vimentin: interaction F (2, 12) = 65.82, *p* < .0001. P21: interaction F (2, 12) = 87.64, *p* < .0001. Specific comparisons indicate that both rhTIMP2 and rhAlaTIMP2 significantly exacerbated the hypoxia‐induced upregulation of fibrosis and senescence markers (*p* < .001 vs. CoCl_2_ alone), with no significant difference observed between the rhTIMP2 and rhAlaTIMP2 groups (*p* > .05). Experiments were repeated in three biological replicates using primary cells isolated from different mice.

To explore whether TIMP2 modulates tubular cell senescence and mitochondrial homeostasis, we employed an in vitro hypoxia‐reoxygenation model using CoCl_2_ to induce stress in PRTCs. In vitro, SA‐β‐gal staining showed that CoCl_2_ significantly induced cellular senescence in WT PRTCs, while TIMP2 knock‐out significantly attenuated SA‐β‐gal activity (Figure 5A). Immunofluorescence analysis showed an increase in γ‐H2AX‐positive signals, a marker of DNA damage, following CoCl_2_ treatment, indicating elevated DNA damage. In contrast, TIMP2‐KO significantly attenuated CoCl_2_‐induced DNA damage (Figure 5B,C). Further analysis revealed that TIMP2‐KO suppressed the expression of dedifferentiation markers (Vimentin, α‐SMA) and ageing‐related proteins (P21, γ‐H2AX) (Figure S6A,B). We further assessed mitochondrial characteristics by staining with MitoTracker red and MitoSOX to evaluate mitochondrial integrity and mitochondrial ROS levels, respectively. As shown in Figure 5D,E, while WT cells exposed to CoCl_2_ exhibited fragmented mitochondria, TIMP2‐KO cells maintained a significantly greater mitochondrial length, indicative of preserved fusion dynamics (Figure 5F,G).

Given that TIMP2 is a secretory protein, we investigated the effects of exogenous TIMP2 on cellular senescence and dedifferentiation using recombinant human TIMP2 (rhTIMP2) to stimulate PRTCs. As shown in Figure 5H,I, rhTIMP2 treatment elevated the expression of senescence markers (P21, γ‐H2AX) and dedifferentiation markers (Vimentin, α‐SMA), indicating that extracellular TIMP2 promotes both tubular cell senescence and loss of the epithelial phenotype. Previous studies suggested that TIMP2 might regulate anti‐angiogenic and anti‐tumoral activities through inhibiting MMP pathways.^36^ To determine whether TIMP2's role in promoting senescence in renal tubular epithelial cells depends on MMP‐inhibitory capabilities, we employed a specific TIMP2 mutant (rhAlaTIMP2) that is structurally modified to lack MMP‐inhibitory function. We then evaluated the effects of both rhTIMP2 and rhAlaTIMP2 at an equivalent concentration of 100 nM on cellular senescence and dedifferentiation in the CoCl_2_‐induced senescence cell model (Figure 5J). Our results showed that both rhTIMP2 and rhAlaTIMP2 exhibited comparable efficacy in promoting cellular senescence and dedifferentiation (Figure 5K,L), indicating that the pro‐senescent function of TIMP2 is independent of its MMP‐inhibitory activity. In addition, no significant variation was found in SASP secretion between rhTIMP2 and rhAlaTIMP2 (Figure 5K,L), supporting the hypothesis that the role of TIMP2 in cellular senescence does not rely on its MMP‐inhibitory function. These findings indicate that TIMP2 may also regulate cellular senescence through non‐MMP‐dependent mechanisms, necessitating further research.

### 2.6 TIMP2 promotes cellular senescence and maladaptive repair through β‐catenin signalling

**FIGURE 6:**
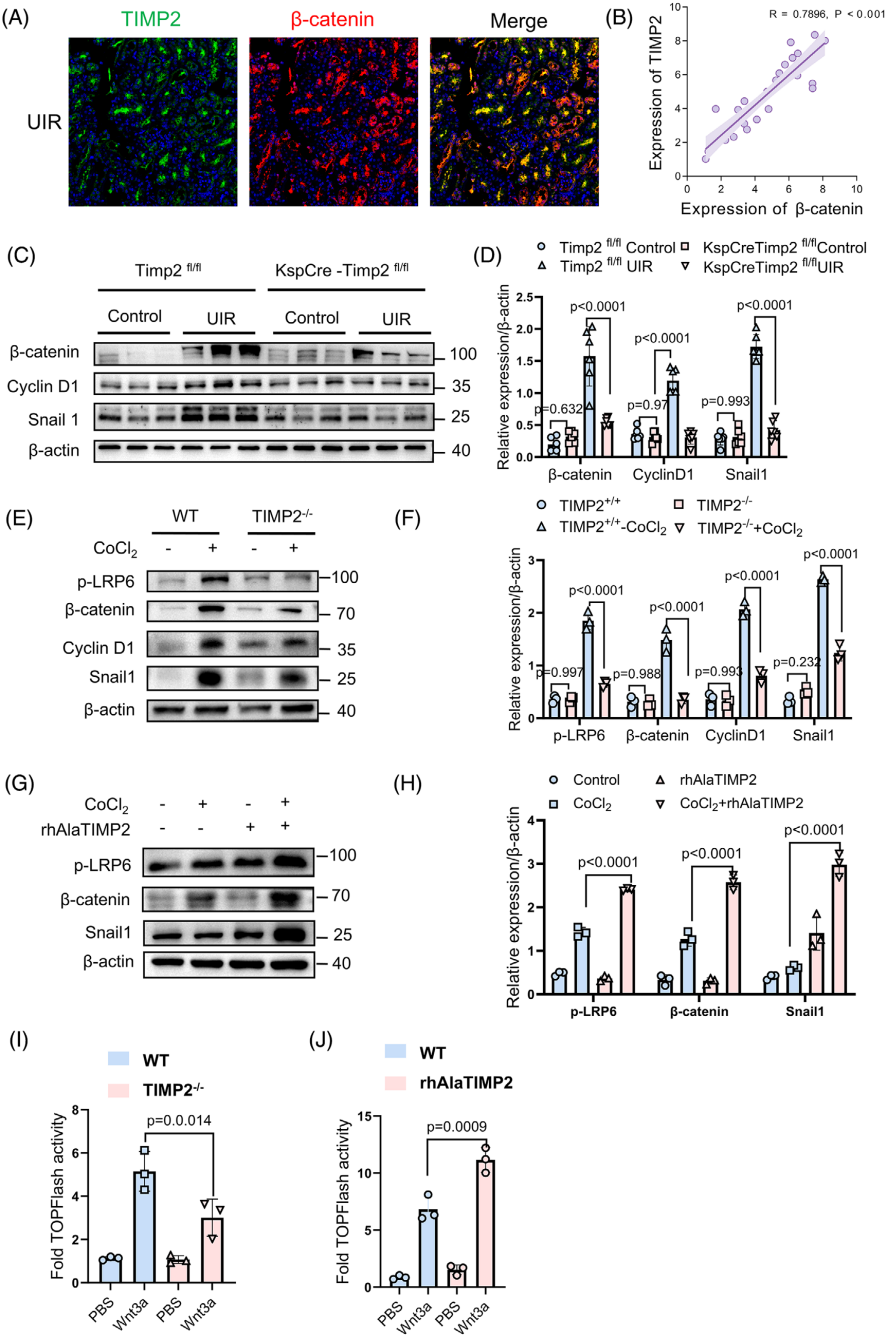
TIMP2 promotes cellular senescence and maladaptive repair through β‐Catenin signalling. (A) Immunofluorescence staining demonstrating the colocalization of TIMP2 (green) and β‐catenin (red) within renal tubules in a mouse model of UIR. Scale bar is 50 µm. (B) Analysis of the correlation between the expressions of TIMP2 and β‐catenin in the UIR mice kidney. The Spearman correlation coefficient (R) and *p* value are shown. (C, D) Western blot detection and quantitative analysis of β‐catenin and Snail1 protein expression differences in WT and Timp2 knockout (*KspCreERT2‐Timp2^fl/fl^*) mice. Each group consisted of *n* = 6. Statistical significance was assessed by two‐way ANOVA followed by Tukey's post hoc test. β‐catenin: interaction F (1, 20) = 135.2, *p* < .0001. CyclinD1: interaction F (1, 20) = 158.3, *p* < .0001. Snail1: interaction F (1, 20) = 312.6, *p* < .0001. Specific comparisons indicate that the injury‐induced activation of Wnt/β‐catenin signalling and downstream targets was significantly suppressed in TIMP2‐deficient kidneys (*p* < .001 vs. WT UIR). (E, F) Western blot analysis comparing the expression levels and quantitative statistics of p‐LRP6 (ser1490), activated β‐catenin, Cyclin D1 and Snail1 in PRTCs treated with and without CoCl_2_ (200 µM) in TIMP2‐KO and WT. Sample size *n* = 3. Data are mean ± SD; two‐way ANOVA test. Experiments were repeated in three biological replicates using primary cells isolated from different mice. (G, H) Western blot detection and quantitative analysis of p‐LRP6, activated β‐catenin, Cyclin D1 and Snail1 expression levels in PRTCs stimulated with Wnt3a and rhAlaTIMP2 in PRTCs. *n* = 3, data are mean ± SD. Statistical significance was assessed by two‐way ANOVA followed by Tukey's post hoc test. p‐LRP6: interaction F (1, 8) = 138.5, *p* < .0001. β‐catenin: interaction F (1, 8) = 205.4, *p* < .0001. Snail1: interaction F (1, 8) = 42.15, *p* < .0001. Specific comparisons indicate that co‐stimulation with rhAlaTIMP2 significantly potentiated the CoCl_2_‐induced activation of Wnt/β‐catenin signalling. Experiments were repeated in three biological replicates using primary cells isolated from different mice. (I) TOPflash reporter assay to measure changes in Wnt/β‐catenin signalling activity in PRTCs from WT or TIMP2^−/−^ mice with or without Wnt3a stimulation. two‐way ANOVA test. (J) TOPflash analysis measuring changes in Wnt/β‐catenin signalling activity in 293T cells under stimulation with AlaTIMP2, Wnt3a and their combined effects. *n* = 3, statistical significance was assessed by two‐way ANOVA followed by Tukey's post hoc test. TopFlash Activity: interaction F (1, 8) = 15.01, *p* = .005; Wnt3a treatment F (1, 8) = 136.2, *p* < .0001; group effect F (1, 8) = 14.05, *p* = .006. Specific comparisons indicate that co‐treatment with rhAlaTIMP2 and Wnt3a elicited a synergistic increase in Wnt/β‐catenin transcriptional activity. Experiments were repeated in three biological replicates using primary cells isolated from different mice.

Previous studies demonstrated that Wnt/β‐catenin signalling drives cellular senescence and fibrosis, thereby accelerating CKD progression.^28^ Kyoto Encyclopedia of Genes and Genomes (KEGG) analysis further revealed a strong association between TIMP2 expression and the Wnt/β‐catenin signalling pathway (Figure 4B). To explore the relationship between TIMP2 and the Wnt/β‐catenin signalling pathway, we focused on β‐catenin, a key protein in the Wnt pathway. Using the UIR model, we examined the colocalization of TIMP2 and β‐catenin expressions. Immunofluorescence staining revealed that TIMP2 expression was colocalized with β‐catenin‐positive tubules (Figure 6A), and there was a positive correlation between the expression levels of TIMP2 and β‐catenin (Figure 6B). Furthermore, in the kidneys of CKD mice induced by UIR, β‐catenin was upregulated, whereas knockout of Timp2 effectively inhibited the activation of β‐catenin (Figure 6C,D). Similarly, the expression of β‐catenin downstream proteins, such as Snail1 and Cyclin D1, was increased in the UIR group of WT mice but significantly reduced in the renal tissues of Timp2‐knockout mice (Figure 6C,D). These findings suggest a crucial role of TIMP2 in regulating the Wnt/β‐catenin signalling pathway. As shown in Figure 6E,F, compared with the control group stimulated with CoCl_2_, TIMP2‐KO suppressed the phosphorylation of the Wnt receptor LRP6, activation of β‐catenin and expression of the downstream target Snail1. In contrast, co‐stimulation with CoCl_2_ and recombinant TIMP2 protein (rhAlaTIMP2) significantly upregulated the expression of β‐catenin and its downstream pathway proteins compared with stimulation with CoCl_2_ alone (Figure 6G,H), indicating that TIMP2 can activate the Wnt/β‐catenin signalling pathway in vitro.

Given that a critical step in the Wnt signalling pathway involves the stabilization and nuclear translocation of β‐catenin, which governs the transcription of downstream target genes, we assessed the regulatory role of TIMP2 using a luciferase reporter assay. PRTCs were transfected with the TopFlash reporter plasmid, which measures β‐catenin/TCF‐mediated transcriptional activity, along with the Fop‐Flash plasmid as a negative control. Under Wnt3a stimulation, β‐catenin‐driven luciferase activity was significantly attenuated in TIMP2‐KO cells compared with controls (Figure 6I). Conversely, in 293T cells, co‐treatment with AlaTIMP2 and Wnt3a elicited a greater increase in TopFlash activity than Wnt3a alone (Figure 6J). These findings indicate that TIMP2 promotes canonical Wnt/β‐catenin signalling.

### 2.7 TIMP2 regulates the Wnt/β‐catenin signalling pathway by promoting LRP6 phosphorylation

**FIGURE 7:**
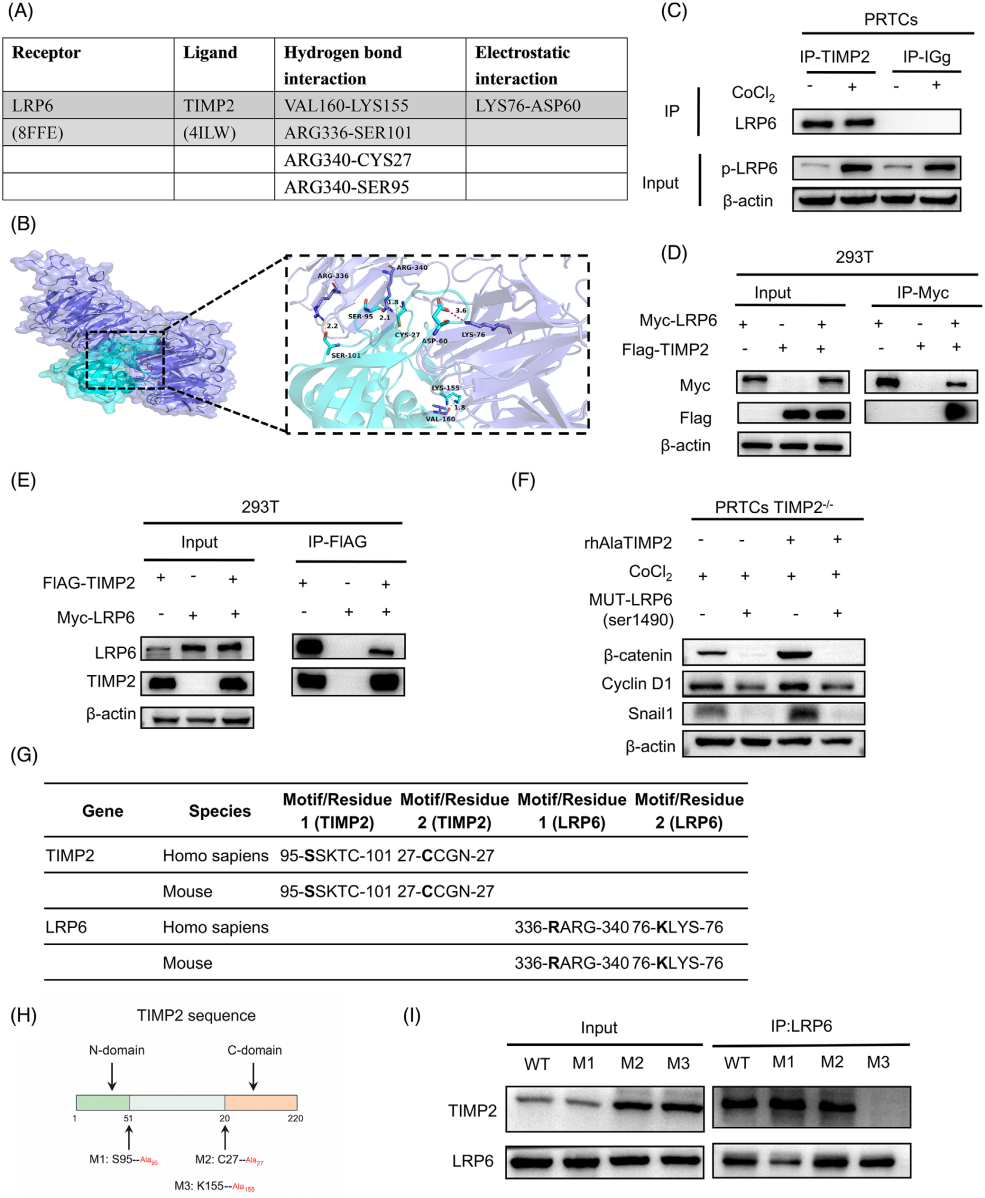
TIMP2 regulates the Wnt/β‐catenin signalling pathway by promoting LRP6 phosphorylation. (A, B) By means of protein–protein interaction analysis in Pymol, all functional residues were identified and classified according to their interactions. In the hydrogen bonding interaction, there are multiple groups of residues used to form hydrogen bonds between LRP6 and TIMP2, such as the hydrogen bond formed by VAL160 of LRP6 and LYS155 of TIMP2. With these interaction forces, the scoring of LRP6‐TIMP2 is ‐608 (you can see the model scoring by opening receptor‐ligand.dat through Notepad), which is a good performance. (C) Co‐immunoprecipitation (Co‐IP) experiment demonstrating an interaction between TIMP2 and LRP6 in PRTCs, as evidenced by protein pulldown using TIMP2 antibody. (D) Co‐IP experiment showing protein pulldown of Myc‐tagged LRP6 with an antibody against FLAG‐tagged TIMP2 in 293T cells transfected with plasmids encoding FLAG‐tagged TIMP2 and Myc‐tagged LRP6. (E) Pulldown of FLAG‐tagged TIMP2 protein using an antibody against Myc‐tagged LRP6. (F) Western blot results showing the expression of phosphorylation of LRP6 and the activation of the downstream Wnt pathway protein β‐catenin, as well as the expression and quantitative statistics of downstream proteins Snail1 and Cyclin D1 in CoCl_2_‐treated PRTCs. (G) Alignment of TIMP2 and LRP6 interface residues across human and mouse, highlighting conserved sites. (H) Diagram of TIMP2 showing mutation sites (S95A, C27A, K155A) used for binding analysis. (I) Co‐IP of LRP6 with TIMP2 mutants in 293T cells. K155A (M3) disrupts TIMP2–LRP6 binding.

We further explored how TIMP2 influences the Wnt/β‐catenin pathway by analyzing the mRNA expression of all 19 Wnt ligands in PRTC stimulated with CoCl_2_. The expression of Wnt ligands, such as Wnt1, Wnt2b, Wnt3a, Wnt6, Wnt7a, Wnt9a and Wnt16, etc., was assessed. TIMP2‐KO did not affect the expression of these ligands (Figure S6C, *p* > .05), suggesting that TIMP2 activates β‐catenin and downstream genes through a mechanism independent of Wnt expression. We hypothesized that secreted TIMP2 enhances Wnt signalling via autocrine or paracrine mechanisms by interacting with membrane receptors. Since Wnt proteins activate downstream signalling by binding to the LRP6 receptor, and previous studies have shown that TIMP2 can bind to LRP family members LRP1 and LRP2 to mediate cellular endocytosis,^37^, ^38^ we speculated that TIMP2 activates Wnt signalling through interaction with LRP6. To validate this hypothesis, PyMOL‐based protein–protein interaction analysis was conducted to identify and classify functional residues (Figure 7A). Notably, VAL160 of LRP6 and LYS155 of TIMP2 participate in hydrogen bonding. The LRP6‐TIMP2 interaction scored −608, indicating strong performance (Figure 7B). Co‐immunoprecipitation (Co‐IP) was also performed on PRTCs stimulated with CoCl_2_, using a TIMP2 antibody to pull down the total protein post‐CoCl_2_ stimulation. Western blotting detected LRP6 as a TIMP2‐interacting protein (Figure 7C), confirming an interaction between TIMP2 and LRP6. To further validate the interaction between TIMP2 and LRP6, Flag‐tagged TIMP2 and Myc‐tagged LRP6 were overexpressed in 293T cells, and Co‐IP confirmed a direct interaction between TIMP2 and LRP6 (Figure 7D,E). Since LRP6 activation depends on phosphorylation at Ser1490,^30^ we next investigated whether this post‐translational modification is required for TIMP2‐LRP6 interaction. To test this hypothesis, PRTCs were transfected with plasmids encoding a phosphorylation‐defective LRP6 mutant (MUT‐pLRP6‐ser1490) that disrupts the critical phosphoracceptor site. Importantly, this mutation abolished the ability of TIMP2 to upregulate Wnt/β‐catenin pathway components (β‐catenin and Snail1), demonstrating that TIMP2‐mediated regulation of Wnt signalling requires LRP6 phosphorylation at this specific residue (Figure 7F). To gain further mechanistic insight, we performed a cross‐species alignment of the key interacting motifs and residues in TIMP2 and LRP6, revealing high evolutionary conservation (Figure 7G). Based on structural predictions, we generated three TIMP2 alanine substitution mutants (S95A, C27A, K155A), targeting critical residues identified at the LRP6–TIMP2 interface (Figure 7H). Co‐IP experiments showed that the K155A substitution (M3) abolished the interaction between TIMP2 and LRP6, compared with wild‐type TIMP2 (Figure 7I), underscoring the essential role of these residues in mediating TIMP2–LRP6 complex formation. Together, these results provide compelling structural, biochemical and functional evidence that TIMP2 interacts with LRP6, promotes its phosphorylation and activates Wnt/β‐catenin signalling. The integrity of specific TIMP2 residues at the interaction interface is indispensable for this regulatory mechanism.

### 2.8 In vivo expression of TIMP2 aggravates β‐catenin‐signalling‐mediated renal fibrosis

**FIGURE 8:**
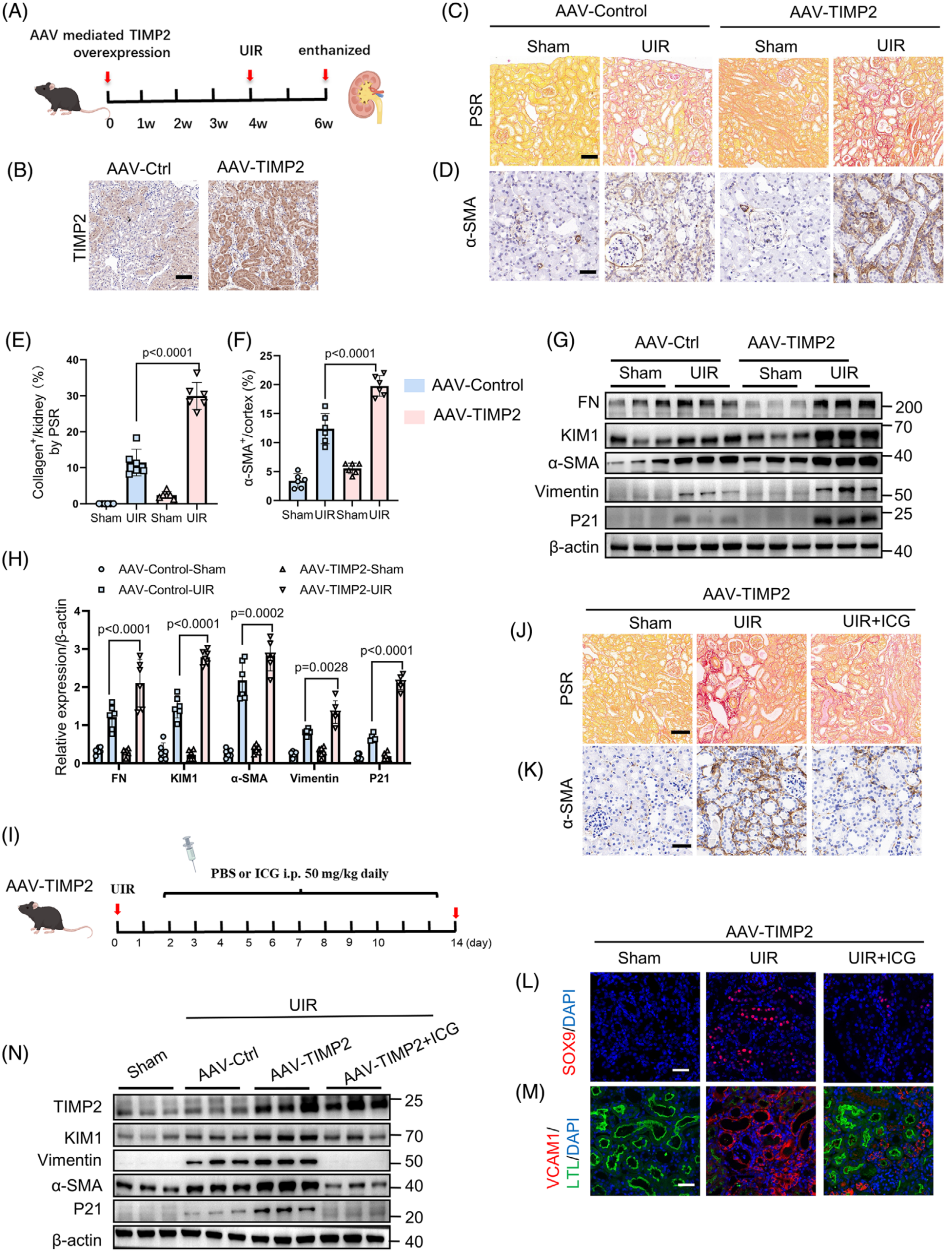
In vivo expression of TIMP2 aggravates β‐catenin‐signalling‐mediated renal fibrosis. (A) Schematic representation of the experimental design. (B) Immunohistochemical staining of TIMP2. (C) PSR staining of renal tissue in mice from the Sham, UIR with AAV‐Control or AAV‐TIMP2 adeno‐associated virus. (D) Immunohistochemical staining of α‐SMA indicating activation of myofibroblasts in renal tissues. Scale bar is 50 µM. (E) Quantification of collagen fibre deposition in the kidney by PSR. *n* = 6, data are mean ± SD. Statistical significance was assessed by two‐way ANOVA followed by Tukey's post hoc test. Fibrosis score: interaction F (1, 20) = 87.54, *p* < .0001; treatment F (1, 20) = 452.1, *p* < .0001; AAV effect F (1, 20) = 112.3, *p* < .0001. Specific comparisons indicate that TIMP2 overexpression significantly exacerbated renal injury and fibrosis compared with the AAV‐Control group. (F) Quantitative analysis of α‐SMA^+^ area/cortex (%). *n* = 6. Data are mean ± SD. Statistical significance was assessed by two‐way ANOVA followed by Tukey's post hoc test. α‐SMA+ area: interaction F(1, 20) = 28.65, *p* < .0001; treatment F(1, 20) = 452.8, *p* < .0001; AAV effect F(1, 20) = 118.4, *p* < .0001. Specific comparisons indicate that the accumulation of myofibroblasts (α‐SMA^+^) was significantly exacerbated in TIMP2‐overexpressing kidneys following UIR. (G, H) Western blot analysis and quantitative statistics of FN, KIM‐1, α‐SMA, Vimentin and P21 expression in renal tissue samples from the AAV‐control and AAV‐TIMP2 groups post‐Sham or UIR surgery. Each group consisted of *n* = 6 samples. Quantitative analysis and expression levels of phosphorylated LRP6, β‐catenin, cyclin D1 and Snail1 in renal tissues. *n* = 6. Data are mean ± SD. Statistical significance was assessed by two‐way ANOVA followed by Tukey's post hoc test. FN: interaction F (1, 20) = 65.42, *p* < .0001. KIM1: interaction F (1, 20) = 108.5, *p* < .0001. α‐SMA: interaction F (1, 20) = 38.75, *p* < .0001. Vimentin: interaction F (1, 20) = 72.14, *p* < .0001. P21: interaction F (1, 20) = 156.8, *p* < .0001. Specific comparisons indicate that TIMP2 overexpression significantly amplified the expression of injury, fibrosis and senescence markers following UIR. (I) Schematic representation of the experimental design. (J) PSR staining to compare and analyze collagen fibre deposition in renal tissues of mice in the Sham group, AAV‐control + UIR group, AAV‐TIMP2 + UIR group and AAV‐TIMP2 + UIR + ICG group. Scale bar is 50 µm. (K) α‐SMA immunohistochemical staining to evaluate activation of interstitial cells and quantify the positively stained areas. Scale bar is 50 µm. (L) Immunofluorescence staining of SOX9 (red) to mark cellular repair processes. Scale bar is 50 µm. (M) Immunofluorescence staining with LTL (green) and VCAM (red) to label the dedifferentiation of renal tubular epithelial cells. Scale bar is 50 microns. (N) Western blot analysis of TIMP2, KIM‐1, Vimentin, α‐SMA and P21 protein expression levels in renal tissues from the aforementioned four groups. Scale bar is 50 µm.

To evaluate the effects of TIMP2 overexpression on the senescence of RTECs and interstitial fibrosis in the AKI‐CKD model, we employed an adeno‐associated virus overexpressing TIMP2 (AAV‐TIMP2) to overexpress TIMP2, aiming to monitor the repair processes in kidney injury (Figure 8A,B). Histopathological and PSR staining indicated that TIMP2 overexpression significantly exacerbated tubular injury and collagen deposition (Figure 8C,E; Figure S7A,D). Immunohistochemistry revealed a significant increase in the fibrosis marker α‐SMA in the TIMP2‐overexpressing group compared with controls following UIR (Figure 8D,F), further supporting the role of TIMP2 in promoting renal fibrosis. Western blot analysis revealed that TIMP2 overexpression in the AKI–CKD model significantly upregulated fibrosis‐ and senescence‐associated proteins, including FN, α‐SMA, KIM‐1, Vimentin and P21 (Figure 8G,H). Immunofluorescence results showed that renal injury marker KIM‐1 and maladaptive repair‐related protein VCAM‐1 were elevated in TIMP2 overexpressing groups post‐UIR surgery (Figure S7B,C,E,F). TIMP2 overexpression significantly elevated the levels of downstream Wnt/β‐catenin signalling proteins, including p‐LRP6, β‐catenin and Snail1, after UIR in mice (Figure S7G,H). These findings suggest that TIMP2 overexpression excessively activates the Wnt/β‐catenin pathway, which accelerates cell senescence and contributes to the progression of AKI‐CKD and renal interstitial fibrosis. Transmission electron microscopy revealed increased mitochondrial damage in renal tissues of mice overexpressing TIMP2, characterized by mitochondrial swelling, disrupted and fragmented cristae and loss of normal ultrastructural integrity (Figure S7I).

To further validate that TIMP2 promotes tubular cell senescence and fibrosis via the Wnt/β‐catenin pathway, mice were intraperitoneally administered the β‐catenin inhibitor ICG‐001 (50 mg/kg),^30^ to both control and TIMP2‐overexpressing mice, using UIR to establish an AKI‐CKD model (Figure 8I). PSR and α‐SMA staining revealed significantly greater collagen deposition in renal tissue of the TIMP2 overexpression group compared with controls after UIR. However, collagen deposition was notably reduced in the inhibitor intervention group (AAV‐TIMP2 + ICG‐001) after UIR (Figure 8J). SOX9^+^ and VCAM^+^ staining was reduced in the ICG‐001 group compared with controls after UIR (Figure 8L,M). Western blot analysis confirmed that ICG‐001 treatment effectively abrogated the TIMP2‐induced upregulation of KIM‐1, Vimentin and α‐SMA (Figure 8N). These findings further reinforce that TIMP2 promotes cell senescence and kidney fibrosis through regulation via the Wnt/β‐catenin signalling pathway.

## 3 DISCUSSION

In this study, we identify TIMP2 as a key mediator of tubular maladaptive repair, showing its pronounced upregulation in renal tubular epithelial cells during the AKI–CKD transition. Genetic ablation of TIMP2, either constitutive or tamoxifen‐induced, alleviated interstitial fibrosis, tubular senescence and sustained kidney injury, thereby delineating a potential therapeutic window even after the acute insult has resolved. Previous studies have primarily regarded TIMP2 as an inhibitor of MMP activity^39^, ^40^; however, our data suggest that TIMP2 also regulates cell senescence and mitochondrial function through MMP‐independent mechanisms. Mechanistically, TIMP2 interacts with the Wnt co‐receptor LRP6, promoting its phosphorylation and subsequent activation of β‐catenin signalling. Previous research has established TIMP2 as a sensitive biomarker of tubular stress in AKI.^41^ Our work provides new evidence that TIMP2 actively promotes cellular senescence, mitochondrial dysfunction and maladaptive repair in renal tubular epithelial cells, fundamentally advancing our understanding of its role in kidney disease pathogenesis.

Accumulating clinical evidence indicates a strong association between AKI and the subsequent development of CKD, with the AKI–CKD transition representing a pivotal inflection point marked by a dynamic interplay between injury and repair mechanisms.^42^ Critical cellular processes—including mitochondrial dysfunction, G2/M cell‐cycle arrest, cellular senescence and myofibroblast activation—have been implicated in maladaptive repair,^43^ yet the underlying molecular regulators remain incompletely understood. In this study, we demonstrate the presence of maladaptive tubular repair after severe AKI across multiple AKI–CKD models and, importantly, identify TIMP2 as a central mediator of this process. TIMP2, produced and secreted by renal tubular epithelial cells, is markedly elevated in urine during early AKI, where it is widely recognized as a clinical biomarker of tubular stress.^20^ Beyond its diagnostic relevance, our data reveal that TIMP2 is functionally involved in driving tubular injury and fibrotic progression. Genetic deletion of TIMP2 significantly enhanced tubular repair, preserved epithelial integrity and attenuated interstitial fibrosis and cellular senescence. These findings suggest that TIMP2 is not only a passive indicator of epithelial stress but also an active contributor to maladaptive repair following AKI.

Increasing evidence highlights the crucial role of cellular senescence in the progression from AKI–CKD.^44^, ^45^ Cellular senescence denotes an irreversible halt in the cell cycle, accompanied by increased production and secretion of pro‐fibrotic and pro‐inflammatory mediators.^44^ Although the role of TIMP2, a component of the senescence‐associated secretory phenotype (SASP), in cellular senescence has been suggested, its underlying mechanisms have remained unclear. Our findings reveal that TIMP2 can promote cell senescence and RTECs dedifferentiation, contributing to CKD development. Our results provide new mechanistic insight into the AKI–CKD transition and point to TIMP2 as a promising therapeutic target for cellular senescence in renal pathology. Mitochondrial dysfunction is not only a hallmark of senescent cells but also a well‐established driver of kidney injury and fibrosis. Strikingly, we observed that TIMP2 deletion preserved mitochondrial ultrastructure and function in the renal tubular compartment, as evidenced by reduced swelling, intact cristae and overall morphological integrity. These ultrastructural improvements in TIMP2‐deficient mice suggest that TIMP2 may contribute to senescence through direct or indirect effects on mitochondrial integrity.

TIMP2 is a secreted protein previously recognized as an endogenous inhibitor of MMPs.^46^ Recent studies have not only highlighted its classical role in inhibiting MMP2 activity^47^ but also discovered that TIMP2 participates in a broad range of biological processes through MMP‐independent pathways, including anti‐tumour activities, brain injury response, myocardial remodelling and fibrosis.^48^, ^49^, ^50^ Given the broad biological functions regulated by TIMP2's non‐proteolytic activities, elucidating its molecular mechanisms in AKI‐CKD is critical. We explored how TIMP2 promotes cellular senescence and fibrosis using recombinant human TIMP2 (rhTIMP2) and its mutant, rhAlaTIMP2. RhAlaTIMP2, designed by substituting key amino acid residues, lacks inhibitory activity against MMPs. This characteristic makes it an ideal tool for studying TIMP2 functions, particularly in MMP‐independent mechanisms. Comparative analysis of rhTIMP2 and rhAlaTIMP2 in a cellular ageing model demonstrated that TIMP2's ability to promote cellular senescence and fibrosis does not depend on its MMP inhibitory capacity. TIMP2 binds directly to the receptor LRP6, activating the Wnt/β‐catenin signalling pathway and, therefore, regulating cellular senescence.

It has been shown that Wnt/β‐catenin signalling can accelerate ageing processes,^51^ highlighting its role in cellular senescence and disease progression. Wnt9a was found to co‐localize with P16‐positive renal tubules, and its expression correlates with renal fibrosis and GFR, suggesting Wnt/β‐catenin pathway involvement in kidney injury.^28^ Previous studies have suggested that the activation of the Wnt/β‐catenin signalling pathway relies on the binding of Wnt ligands to their receptors. Remarkably, our study uncovers a non‐canonical mechanism whereby TIMP2, a secreted protein previously recognized primarily for its MMP‐inhibitory activity, can directly interact with LRP6 to trigger its phosphorylation and downstream β‐catenin activation, independent of Wnt ligands. This finding challenges the conventional paradigm of Wnt signalling regulation and broadens the scope of molecular inputs capable of modulating this critical pathway.

Our study has some limitations. First, the findings are derived solely from preclinical murine models, and the clinical relevance of TIMP2 in human kidney injury remains unvalidated. Human renal specimens were not analyzed to correlate TIMP2 expression levels with disease severity, histopathological changes, or functional outcomes in patients with AKI or CKD. Second, while this study primarily focuses on the role of cellular senescence in CKD, it is important to note that many of the gene products implicated in senescence are also involved in broader stress responses.

In summary, we have provided compelling evidence showing that TIMP2 promotes senescence and maladaptive repair in renal tubular cells through activation of the Wnt/β‐catenin signalling pathway, leading to renal interstitial fibrosis. By elucidating the mechanistic role of TIMP2, our research not only enhances our understanding of its function in AKI–CKD transition but also paves the way for translating its potential as a biomarker into effective therapeutic interventions.

## 4 MATERIALS AND METHODS

Detailed protocols for all methods are provided in the Supporting Information Materials.

### 4.1 Animals studies

Male mice were exclusively used in this AKI study, as females display greater resistance to renal ischemia–reperfusion injury and cisplatin‐induced nephrotoxicity. Whether the findings in males are applicable to females remains uncertain. Male mice aged 8–12 weeks were maintained under a standard 12 h light/dark cycle with free access to food and water. Timp2^flox/flox^ mice were crossed with either Ksp‐Cre transgenic mice (expressing Cre recombinase under the cadherin‐16 promoter) or CreERT2 mice to generate two types of TIMP2 conditional knockouts (GemPharmatech, Jiangsu, China). The LoxP sites were inserted between exons 1 and 4 of the TIMP2 gene by GemPharmatech. For CreERT2 induction, tamoxifen dissolved in corn oil was administered intraperitoneally. Littermates were used as controls in all experiments.

In brief, mice were anesthetized and subjected to unilateral renal ischemia by clamping the left renal pedicle via a retroperitoneal approach for 30 min at 37°C, as previously described.^52^ After surgery, 1 mL of warm saline (37°C) was administered intraperitoneally for volume replacement. Sham controls underwent kidney exposure without vascular clamping. The UUO model was established by ligating the left ureter. Cisplatin nephropathy was induced by weekly intraperitoneal injections of cisplatin (8 mg/kg; Sigma‐Aldrich, St. Louis, MO, USA) in PBS for 4 consecutive weeks. All experiments were performed in a blinded manner with respect to genotype and treatment. Animal studies were approved by the Animal Care Committee of Zhongnan Hospital, Wuhan University, and conducted in accordance with institutional guidelines (approval no. WP20220489).

Genotyping was performed on tail DNA by standard PCR employing the following primer sets:

*KspCreERT2*:AGTCTAGTGGGACTGGGGAC, CCTCCTCATCCTCTCCCACA; *KspCre*:GGGCAGTCTGGTACTTCCAAGCT, ACTGAGTGCCTACTAACCAGCACC; *Timp2^flox/flox^*:ACCGGCTGTCCATCAACCCTAC, AATCCTTTGCTGACAAGGGTGAAG.

### 4.2 GFR measurement in conscious animals

Transcutaneous GFR measurement was conducted as previously reported.^53^ One day before the experiment, the flank region was shaved and depilated to optimize optical detection. Mice were briefly anesthetized with isoflurane for device placement (MediBeacon GmbH, Mannheim, Germany), which was secured with elastic gauze. After recovery from anaesthesia (3–5 min), baseline fluorescence was recorded, followed by intravenous injection of FITC‐sinistrin (0.7 mg/kg in PBS, 35 mg/mL). Real‐time GFR was monitored continuously for ∼1 h and analyzed with MB Studio v.22 (MediBeacon GmbH) using a three‐compartment model with linear baseline correction. To reduce variability, measurements were performed at a consistent time of day under controlled temperature and light conditions.

### 4.3 Analysis of human kidney spatial multi‐omics atlas

To characterize the spatial distribution of TIMP2 in human kidney tissue, we utilized the high‐resolution spatial multi‐omics atlas from a recent study by Abedini et al.^31^ The data were accessed via the Susztak Lab Spatial Atlas portal (https://susztaklab.com/samui/). Specifically, we analyzed the spatial transcriptomics data (Visium and CosMx) from kidney biopsy samples HK2671, HK2770, HK2844, HK2852, HK2871, HK2873 and HK3035. Quality control and data processing were performed as described in the original study.

### 4.4 Statistical analyses

All results are based on at least three independent experiments. Statistical analysis was conducted using Prism 10 software (GraphPad Software, Inc.). The data are presented as mean  ±  SD. Differences between groups were analyzed by one‐way ANOVA with Tukey's post hoc test or, for multiple factors, by two‐way ANOVA. A *p*‐value > .05 was considered not significant; *p* < .05; *p* < .01; *p* < .001 was considered statistically significant.

## AUTHOR CONTRIBUTIONS

**Dongxue Xu**: Writing—original draft; project administration; investigation. **Haichuan Yu**: Project administration and software. **Jingjing Pang**: Project administration and investigation. **Xiaoyu Zhang**: Investigation and software. **Jun Jiang**: Software. **Yiming Li**: Visualization and funding acquisition. **Zhiyong Peng**: Writing—review & editing; Writing—original draft and Funding acquisition.

## CONFLICT OF INTEREST STATEMENT

The authors declare no conflict of interest.

## ETHICS STATEMENT

All animal experiments were approved by the Animal Care Committee at Zhongnan Hospital of Wuhan University, and conducted following the procedures of the Animal Protocol Committee of Wuhan University (approval number: WP20220489).

## Supporting information



Supporting Information

## Data Availability

All RNA‐seq data have been deposited in the NCBI database under BioProject ID: PRJNA1185443.
