# Supplementary material for: TIMP2 promotes AKI‐CKD transition by inducing tubular maladaptive repair and cell senescence via targeting Wnt/β‐catenin signalling
Source: Clin Transl Med. 2026 Jan 28;16(2):e70605. doi: 10.1002/ctm2.70605 (PMC12852052; doi:10.1002/ctm2.70605)
Supplement: Supplementary file 1 — Supporting Information [file CTM2-16-e70605-s001.pdf]

TIMP2 promotes AKI-CKD transition by inducing tubular maladaptive repair and cell senescence via targeting Wnt/β-catenin signaling

Running title: TIMP2 regulates cell senescence in AKI-CKD

Dongxue Xu[1,2#], Haichuan Yu[1,2#], Jingjing Pang[1,2#], Xiaoyu Zhang[1,2], Jun Jiang[1,2], Yiming Li[1,2]*, Zhiyong Peng[1,2,3]*

[1.] Department of Critical Care Medicine, Zhongnan Hospital of Wuhan University, Wuhan 430071, China;

[2.] Clinical Research Center of Hubei Critical Care Medicine, Wuhan 430071, China;

[3.] Intensive Care Unit of the second affiliated Hospital of Hainan Medical College, Haikou, Hainan 570100, China

# Dongxue Xu, Haichuan Yu, and Jingjing Pang contributed equally to this work

* Corresponding authors: Yiming Li, MD, PhD and Zhiyong Peng, MD, PhD

The email of Zhiyong Peng: zn001590@whu.edu.cn

Supplemental Methods

Supplemental Figure 1.

Supplemental Figure 2.

Supplemental Figure 3.

Supplemental Figure 4.

Supplemental Figure 5.

Supplemental Figure 6.

Supplemental Figure 7.

Methods

Reagents

$CoCl_2$ (HY-P7117), Wnt3a (HY-P70453A), ABT263 (HY-10087) and ICG-001 (HY-14428) were purchased from Med Chem Express (MCE, Monmouth Junction, NJ, USA).

Anti-TIMP2 (sc-21735) was purchased from Santa Cruz Biotechnology. LTL-FITC (FL-1321-2) was purchased from Vector Laboratories. Anti-SOX9 (ab185230), anti-VCAM1 (ab134047), anti-P21 (ab10821) and Anti-β-catenin (ab32572) antibodies were purchased from Abcam (Cambridge, UK). Anti-KIM1 (AF1817) and anti-TIMP2 (AF971) were purchased from R&D System (Minneapolis, MN, USA). Anti-γ-H2AX (#9718), anti-LRP6 (#2568), Anti-Fibronectin (#26836), anti-Cyclin-D1 (#55506), anti-Snail1(#3879) antibodies were purchased from Cell Signaling Technology (CST, Danvers, MA, USA). Vimentin (AP-10366) and anti-F4/80 (AP-28463-1) antibodies were purchased from Proteintech. Anti-AQP2 (29386-1-AP), anti-Cdh16 (15107-1-AP), anti-KIM1 (30948-1-AP) antibodies were purchased from Proteintech (Wuhan, China).

Primary mouse renal tubular cells

Primary murine renal tubules were isolated following a previously established protocol with modifications [1]. Murine kidneys were removed, washed with phosphate-buffered saline (PBS), decapsulated, and sliced into four to five slices. Slices of each kidney were transferred to a 2-ml reaction tube containing 2 mg/ml collagenase type II in incubation solution (48 μg/ml trypsin inhibitor, 25 μg/ml DNase I, 140 mM NaCl,

0.4 mM KH_2PO_4, 1.6 mM $K_2HPO_4 \cdot 3\ H_2O$, 1 mM $MgSO_4 \cdot 7\ H_2O$, 10 mM $CH_3COONa \cdot 3\ H_2O$, 1 mM α-ketoglutarate and 1.3 mM Ca-gluconate) and digested for 5 min at 37 °C and 850 rpm.

Primary cells isolated from C57BL/6 mice were tested for mycoplasma contamination by Procell (Wuhan, China) using qPCR. All samples were confirmed negative.

Primary tubular cells were treated with 200μM $CoCl_2$ induced hypoxic stress for the indicated duration[2].

Cell preparation and stimulation

Human embryonic kidney 293 cells (HEK293) were provided by Pricella (China). HEK293 were maintained in high glucose Dulbecco's Modified Eagle Medium (DMEM) supplemented with 10% fetal bovine serum (FBS, Gibco, Grand Island, NY, USA) and 1% streptomycin/penicillin. The cells were maintained in a humidified incubator at 37 °C under 5% CO_2.

Expression and purification of recombinant TIMP2 Protein

The pcDNA3.1B plasmids containing rhTIMP2, rhAlaTIMP2 with a Myc-His tag was constructed and transfected into HEK293T cells using polyethyleneimine (PEI, Yeason Biotechnology, 40816ES02, Shanghai, China). Sixteen hours post-transfection, the medium was replaced with serum-free DMEM (Thermo Fisher Scientific, 10564011, Carlsbad, CA, USA). Culture supernatants containing secreted TIMP2 proteins were collected on the sixth day after transfection and concentrated by ultrafiltration. Recombinant human TIMP2 (rhTIMP2) and its mutant rhAlaTIMP2, were purified using HisPur Ni-NTA resin (Thermo Fisher Scientific, 88221, Waltham, MA, USA)

via histidine tag affinity, eluted with 500 mM imidazole, and dialyzed into PBS (pH 7.4) using a 3 kDa molecular weight cutoff filter (Millipore, UFC900396, St. Louis, MO, USA). The purified recombinant TIMP2 proteins were aliquoted and stored at -80°C until use.

Molecules docking

The X-ray crystal structures of LRP6(8FFE) and TIMP2(4ILW) were retrieved from the Protein Data Bank. To ensure the accuracy of the docking results, the protein was prepared by the AutoDockTools-1.5.7, and the water molecules were manually eliminated from the protein and the polar hydrogen was added. Docking Web Server (GRAMM) was used for protein-protein docking. The resulting protein-protein complex was also manually optimized by removing water and adding polar hydrogen by the AutoDockTools-1.5.7. Finally, the protein-protein interactions were predicted and the protein-protein interaction figure was generated by PyMOL. The LRP6 protein is represented as a slate cartoon model, TIMP2 protein is shown as a cyan cartoon model, and their binding sites are shown as the corresponding-colored stick structure. When focusing on the binding region, the binding site is then shown as a colour of the protein to which it belongs.

AAV9 Construction and Tail Vein Injection

To induce overexpression of Timp2 in mice RTECs, we retrieved the coding sequence of Timp2 (NM_011594) from NCBI and cloned it into the GV736 vector (Ksp-cadherin p-MCS-3flag-T2A-EGFP, specifically expressed in mice renal tubules). Recombinant AAV9 was generated in AAV-293 cells, purified, and then injected into the tail vein of

mice for delivery to the kidneys.

Histology Assessment

Kidney sections were also subjected to Masson-Trichrome staining for assessing collagen deposition and fibrotic lesions. Kidney interstitial fibrosis score was assessed according to the following scale: 0, no evidence of interstitial fibrosis; 1, <25% involvement; 2, 25% to 50% involvement; and 3, >50% involvement[3]. The scale for each animal was reported as the mean of 20 random high-powered (×400) fields per section.

Renal tubular damage was evaluated according to Paller's method[4]. Briefly, five fields were randomly observed, and morphological damage (epithelial necrosis, luminal necrotic debris, and tubular dilation) was quantified using the following scale: none = 0; <10% = 1; 11–25% = 2; 26–75% = 3; and >75% = 4. Nuclei were stained with DAPI (H-1500, Newark, Vector, CA, USA). Fluorescence was visualized and photographed under fluorescence microscopy (Olympus, Shinjuku-ku, Tokyo, Japan).

Bulk RNA-sequencing (RNA-seq) analysis

The fresh renal cortices of WT, TIMP2-knockout, WT + UIR and TIMP2- knockout + UIR mice were immersed in TRIZOL buffer, quick-frozen in liquid nitrogen, and then sent to (Novogene Co., Ltd, China) in dry ice packaging for RNA-seq analysis, and the resulting data were deposited in the SRA database.

Plasmid construction

The mutants, The LRP6 was generated through a two-round PCR amplification procedure using specific primers. Subsequently, all of these DNA fragments were

cloned into the BamHI and XhoI sites of the modified lentiviral vector pBOB, which lacked a tag or included Flag/HA/GFP tags. The Exo III-assisted ligation- independent cloning method was employed for subcloning purposes. To validate the constructed plasmids, they underwent thorough verification via DNA sequencing.

WB analysis and IP

Total protein was extracted from PRTCs. Equal amounts of proteins were separated by sodium dodecyl sulfate–polyacrylamide gel electrophoresis (SDS–PAGE) and transferred to a nitrocellulose membrane (Millipore). After blocking with 5% bovine serum albumin (BSA), the membrane was incubated with a specific antibody. All WB experiments were repeated three times. For immunoprecipitation (IP), whole-cell lysates were lysed in IP buffer containing 1.0% (vol/vol) Nonidet P40, 50 mM Tris–HCl (pH 7.4), 50 mM EDTA, 150 mM NaCl, and a protease inhibitor. After 15 min of centrifugation at 12,000 × g and 4 °C, the protein concentrations in the lysates were measured with a bicinchoninic acid assay (Pierce, Rockford, IL), and the supernatants were collected and incubated overnight with protein G Plus-Agarose IP reagent with a specific antibody. The beads were washed five times with IP buffer, and the immunoprecipitants were eluted by boiling with 1% (wt/vol) SDS sample buffer.

Immunofluorescence

For immunofluorescent staining of mouse kidneys, renal sections were deparaffinized and rehydrated. Tris-EDTA buffer (pH 9.0, Servicebio, China) was used for antigen retrieval by boiling for 10 min in a microwave. Primary cells growing on glass slides were washed with PBS twice and then fixed in 4% paraformaldehyde for 15 min at

room temperature. Following permeabilization with 0.02% Triton X-100 (Sigma–Aldrich, St. Louis, USA) in PBS for 45 min at room temperature, the tissue sections and cell slides were blocked in 10% donkey serum for 1 h at room temperature. Then, the slides were incubated with primary antibodies diluted in PBS overnight at 4 °C. Alexa-conjugated secondary antibodies were used for fluorescence detection. Finally, the slides were mounted in a mounting medium with DAPI (ab104139, Abcam, USA).

Transmission electron microscopy

Cells or renal cortical tissue blocks (1 mm^3) were fixed with 2.5% glutaraldehyde at 4˚C overnight. The prefixed samples were washed with PBS and postfixed with 1% osmium tetroxide for 2 hours at room temperature. The samples were then washed again, dehydrated through an ascending alcohol gradient, and finally embedded. Ultrathin sections (100 nm) were cut with a Leica EM UC7 ultramicrotome (Leica Microsystems, Wetzlar, Germany), stained with uranyl acetate (20 min) and lead citrate (12 min), and were viewed with a Tecnai G2 Spirit transmission electron microscope (FEI, Hillsboro, OR, USA).

TOP-Flash luciferase reporter assay

The luciferase assay was conducted using a dual luciferase assay system kit (E1910; Promega, Madison, WI).

Mitochondrial ROS measurement

Mitochondrial ROS levels were assessed using the MitoSOX™ Red mitochondrial superoxide indicator (Invitrogen, Carlsbad, CA, USA). Briefly, 50 μg of MitoSOX Red was dissolved in 13 μL of DMSO (MP Biomedicals, Solon, OH, USA) to prepare a

5 mM stock solution. The stock solution was then diluted 1,000-fold in Hank's balanced salt solution (HBSS) to obtain a 5 μM working solution. Cells were incubated with 1 mL of the MitoSOX working solution at 37 °C in a humidified atmosphere containing 5% CO_2 for 10 minutes, protected from light. After incubation, cells were collected and washed three times with warm HBSS. Mitochondrial ROS levels were subsequently analyzed by fluorescence microscopy.

Quantitative real-time PCR

The total RNA was isolated from the frozen renal cortices of mice using TRIzol reagent (Invitrogen, USA). cDNA was then synthesized using PrimeScript RT Reagent Kit (Takara, Japan). The target genes were amplified (SYBR Green Kit, Takara, Japan) and quantified in a real-time fluorescence-based quantitative PCR machine (Illumina Eco, USA). The relative gene expression was quantified by the $2^{-\triangle\triangle CT}$ method, and glyceraldehyde-3-phosphate dehydrogenase (GAPDH) was used as a control for normalization. The primers as follows:

Mouse-Wnt-1	CGAGAGTGCAAATGGCAATTCCG
	GATGAACGCTGTTTCTCGGCAG
Mouse-Wnt-2	AGGATGCCAGAGCCCTGATGAA
	CGCCTGTTTTCCTGAAGTCAGC
Mouse-Wnt-2b	CCATTACGGTGTTCGCTTTGCC
	CAGCTTCAGGAATCTCCGAACAG
Mouse-Wnt-3	AGGATGCCAGAGCCCTGATGAA
	AAGTCGCCAATGGCACGGAAGT
Mouse-Wnt-3a	CCGCTCAGCTATGAACAAGCAC
	AAGTCGCCAATGGCACGGAAGT
Mouse-Wnt-4	GAGAACTGGAGAAGTGTGGCTG
	CTGTGAGAAGGCTACGCCATAG
Mouse-Wnt-5a	GGAACGAATCCACGCTAAGGGT
	AGCACGTCTTGAGGCTACAGGA
Mouse-Wnt-5b	GCTACCGCTTTGCCAAGGAGTT
	CATTTGCAGGCGACATCAGCCA

Mouse-Wnt-6	TTTCCGACGCTGGAACTGCTCC
	CCTGACAACCACACTGTAGGAG
Mouse-Wnt-7a	TTCGCCAAGGTCTTCGTGGATG
	TACAGGAGCCTGACACACCATG
Mouse-Wnt-8a	GGTGACTTGGAAAACTGCGGCT
	CCAAACTGTCCACGAAGAGTCTG
Mouse-Wnt-8b	CGGAGACTTTGACAACTGTGGC
	CTGCTTGGAAATTGCCTCTCCG
Mouse-Wnt-9a	AGTGCCAGTACCAGTTCCGCTT
	GAGATGGCGTAGAGGAAAGCAG
Mouse-Wnt-9b	AGAGAGGAAGCAAGGACCTGAG
	GAGAGCTGCTTCCAACAGGTAC
Mouse-Wnt-10a	GCTCCTGTTCTTCCTACTGCTG
	ATGTCAGGCACACTGTGTTGGC
Mouse-Wnt-10b	ACCACGACATGGACTTCGGAGA
	CCGCTTCAGGTTTTCCGTTACC
Mouse-Wnt-11	GCCTGTGAAGGACTCAGAACTTG
	AGCTGTCACTGCCGTTGGAAGT
Mouse-Wnt-16	CCCTCTTTGGCTATGAGCTGAG
	GGTGGTTTCACAGGAACATTCGG
Mouse-IL-6	TACCACTTCACAAGTCGGAGGC
	CTGCAAGTGCATCATCGTTGTTC
Mouse-TGFb1	TGATACGCCTGAGTGGCTGTCT
	CACAAGAGCAGTGAGCGCTGAA
Mouse-TIMP2	GGGAATGACATCTATGGCAACCC
	GGGCCGTGTAGATAAACTCGATG

References

1. Xu, D., et al., *TIMP2 protects against sepsis-associated acute kidney injury by cAMP/NLRP3 axis-mediated pyroptosis.* Am J Physiol Cell Physiol, 2024. **326**(5): p. C1353-c1366.
2. Gui, Y., et al., *Fibroblast expression of transmembrane protein smoothened governs microenvironment characteristics after acute kidney injury.* J Clin Invest, 2024. **134**(13).
3. Ots, M., et al., *Effects of combination therapy with enalapril and losartan on the rate of progression of renal injury in rats with 5/6 renal mass ablation.* J Am Soc Nephrol, 1998. **9**(2): p. 224-30.
4. Paller, M.S., J.R. Hoidal, and T.F. Ferris, *Oxygen free radicals in ischemic acute renal failure*

in the rat. J Clin Invest, 1984. **74**(4): p. 1156-64.

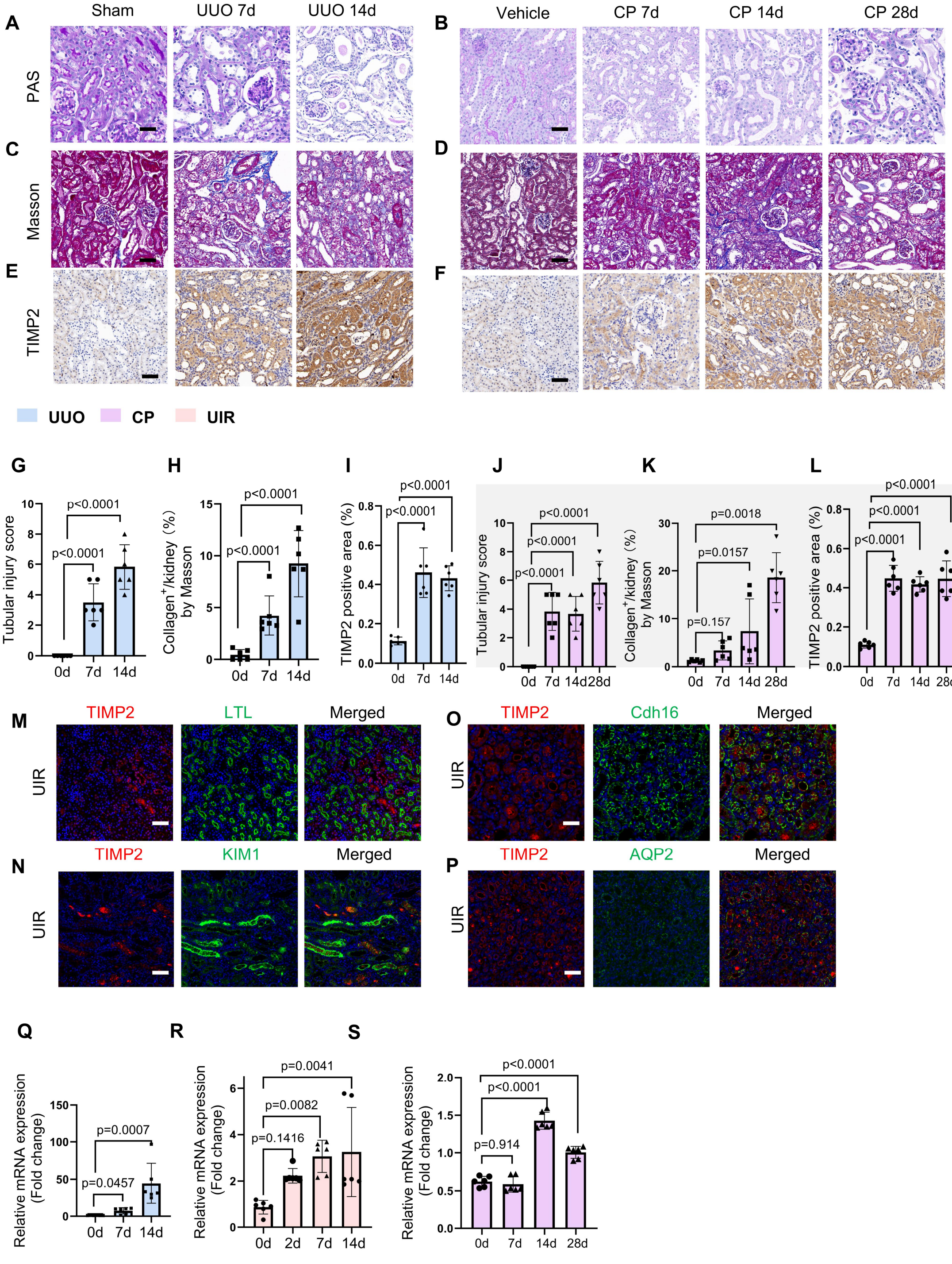

Supplementary Figure 1 TIMP2 Expression is Upregulated in UUO and Cisplatin-Induced Models of AKI-CKD

A-B: PAS staining of kidney tissues after UUO and CP. The scale bar is 50 μm. **C-D:** Masson staining of kidney tissues from UUO and CP. The scale bar is 50 μm. **E-F:**

Immunohistochemical staining demonstrates TIMP2 expression in the renal tubules. The scale bar is 50 μm. **G:** Quantitative injury scoring on the basis of PAS stainings after UUO (n=6, data are mean ± s.d, one-way ANOVA test). **H:** Quantification of collagen fiber deposition in kidney by Masson after UUO (n=6, data are mean ± s.d, one-way ANOVA test). **I:** Quantification of the percentage of TIMP2 in kidney tubules after UUO (n=6, data are mean ± s.d, one-way ANOVA test). **J:** Quantitative injury scoring on the basis of PAS staining after CP (n=6). **K:** Quantification of the percentage of TIMP2 in kidney tubules after CP (n=6, data are mean ± s.d, one-way ANOVA test). **L:** Quantification of the percentage of TIMP2 in kidney tubules after CP (n=6, data are mean ± s.d, one-way ANOVA test). Red signal indicates TIMP2. **M**: Co-staining with LTL (green) shows TIMP2 localization in proximal tubules. **N**: Co-staining with KIM-1 (green) confirms expression in injured proximal tubules. **O**: Co-staining with Cdh16 (green) reveals robust TIMP2 expression in the distal nephron. **P**: Co-staining with AQP2 (green) further confirms TIMP2 expression in the collecting ducts. Scale bar = 20 μm. The widespread co-localization with Cdh16 and AQP2 validates the use of the Ksp-Cre driver. **Q:** Quantitative PCR (qPCR) analysis of TIMP2 mRNA expression levels in mice UUO model (n=6, data are mean ± s.d, one-way ANOVA test). **R:** qPCR analysis of TIMP2 mRNA expression levels in mice UIR model (n=6, data are mean ± s.d, one-way ANOVA test). **S:** qPCR analysis of TIMP2 mRNA expression levels in mice CP model (n=6, data are mean ± s.d, one-way ANOVA test).

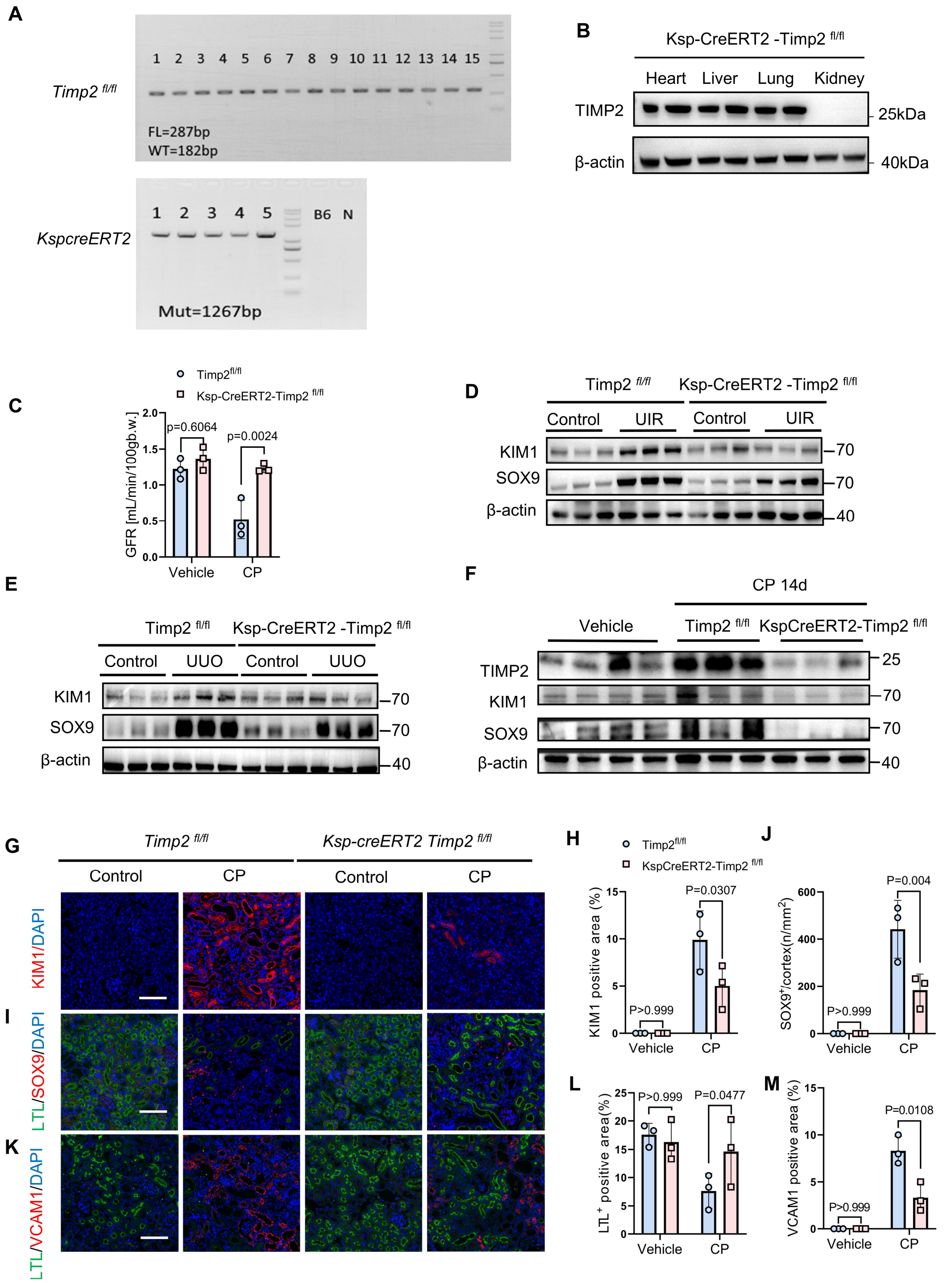

Supplementary Figure 2 TIMP2 Deletion Reduce Maladaptive Tubular Repair After Injury in Cisplatin and UUO Models

A: Garose gel electrophoresis depicting bands for TIMP2$^{fl/fl}$ and Ksp-creERT2. Specific bands indicate the presence of loxP sites flanking the TIMP2 gene and cre recombinase

under the control of the cdh16 promoter, confirming the genetic modification in the mouse model. **B:** Western blot analysis of TIMP2 protein expression in multiple organs (Heart, Liver, Lung, and Kidney) harvested from *Ksp-creERT2; TIMP2*$^{flox/flox}$. β-actin was used as a loading control. The blot demonstrates that TIMP2 protein is specifically ablated in the kidney lysate while remaining abundantly expressed in extra-renal tissues, confirming the tissue specificity of the genetic knockout. **C**: Dynamic change in glomerular filtration rate after CP in *Ksp-creERT2; TIMP2*$^{flox/flox}$ *and TIMP2*$^{flox/flox}$ mice (n=3, data are mean ± s.d, one-way ANOVA test). **D-F:** Western blot of TIMP2, KIM1, and SOX9 protein levels in *TIMP2*$^{flox/flox}$ and Timp2 knockout (Ksp-creERT2; TIMP2$^{flox/flox}$) mice after tamoxifen injection. expression of the kidney injury molecule-1 (KIM-1) in *TIMP2*$^{flox/flox}$ and Timp2 knockout (*Ksp-creERT2; TIMP2*$^{flox/flox}$) mice within UIR, UUO and CP model. **G:** Immunofluorescence staining showing the expression of KIM-1 in *TIMP2*$^{flox/flox}$ and Timp2 knockout (*Ksp-creERT2; TIMP2*$^{flox/flox}$) mice within CP model. **H:** Quantitative analysis of KIM1^{+} area/cortex (%) (n=3, data are mean ± s.d, one-way ANOVA test). **I-J**: Immunofluorescence staining and quantitative analysis of SOX9 across *TIMP2*$^{flox/flox}$ and Timp2 knockout mice subjected to Sham and CP treatments. The scale bar is 50 μm (n=3, data are mean ± s.d, one-way ANOVA test). **K**: Immunofluorescence staining displaying the dual labeling of LTL and SOX9. The scale bar is 50 μm. **L:** Quantitative analysis of LTL^{+} area/cortex (%) (n=3, data are mean ± s.d, one-way ANOVA test). **M:** Quantitative analysis of SOX9^{+} area/cortex (%) (n=3, data are mean ± s.d, one-way ANOVA test). Statistical significance is indicated by * $p < 0.05$, ** $p < 0.01$, and *** $p < 0.001$.

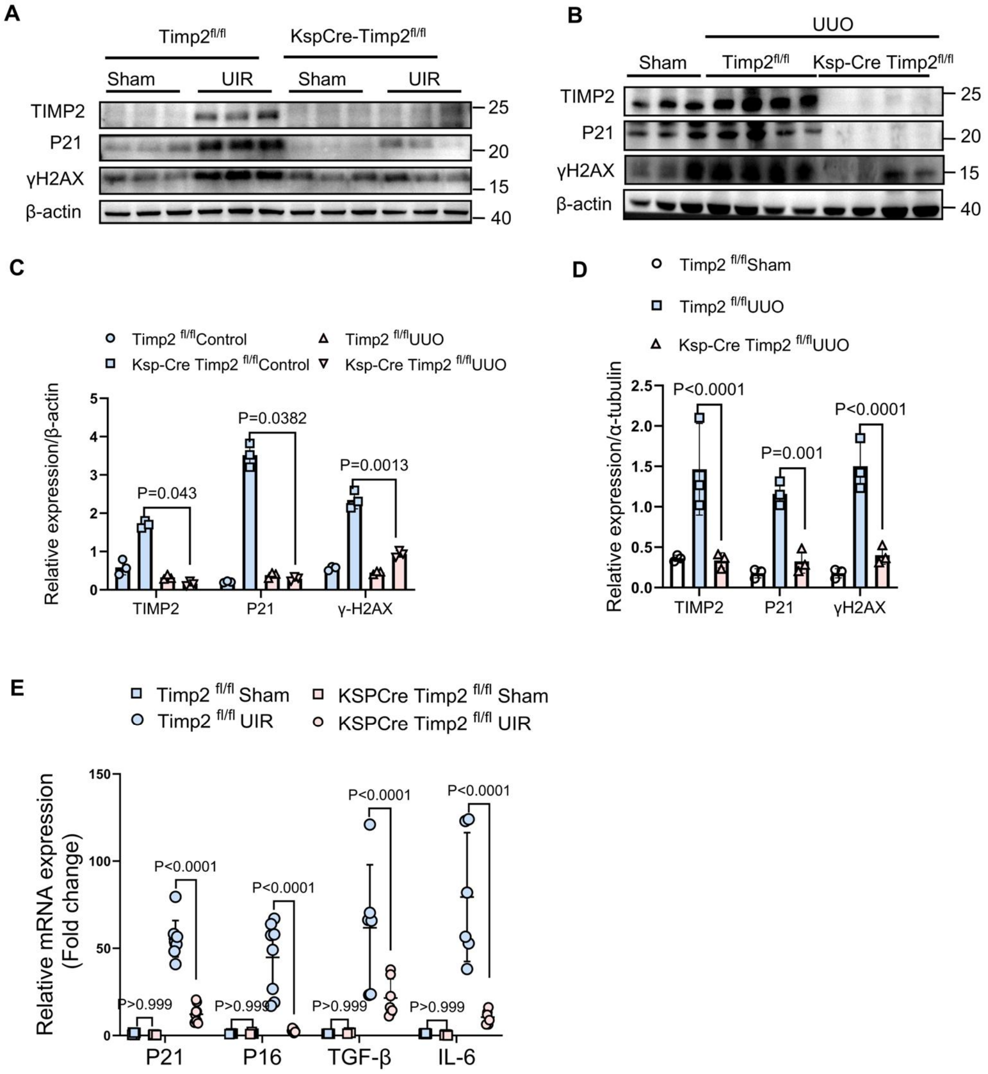
A
Timp2fl/fl
KspCre-Timp2fl/fl
Sham
UIR
Sham
UIR
TIMP2
P21
γH2AX
β-actin
25
20
15
40
B
UUO
Sham
Timp2fl/fl
Ksp-Cre Timp2fl/fl
TIMP2
P21
γH2AX
β-actin
25
20
15
40
C
Timp2 fl/fl Control
Ksp-Cre Timp2 fl/fl Control
Timp2 fl/fl UUO
Ksp-Cre Timp2 fl/fl UUO
Relative expression/β-actin
P=0.043
P=0.0382
P=0.0013
TIMP2
P21
γ-H2AX
D
Timp2 fl/fl Sham
Timp2 fl/fl UUO
Ksp-Cre Timp2 fl/fl UUO
Relative expression/α-tubulin
P<0.0001
P=0.001
P<0.0001
TIMP2
P21
γH2AX
E
Timp2 fl/fl Sham
Timp2 fl/fl UIR
KSPCre Timp2 fl/fl Sham
KSPCre Timp2 fl/fl UIR
Relative mRNA expression (Fold change)
P<0.0001
P<0.0001
P<0.0001
P<0.0001
P>0.999
P>0.999
P>0.999
P>0.999
P21
P16
TGF-β
IL-6

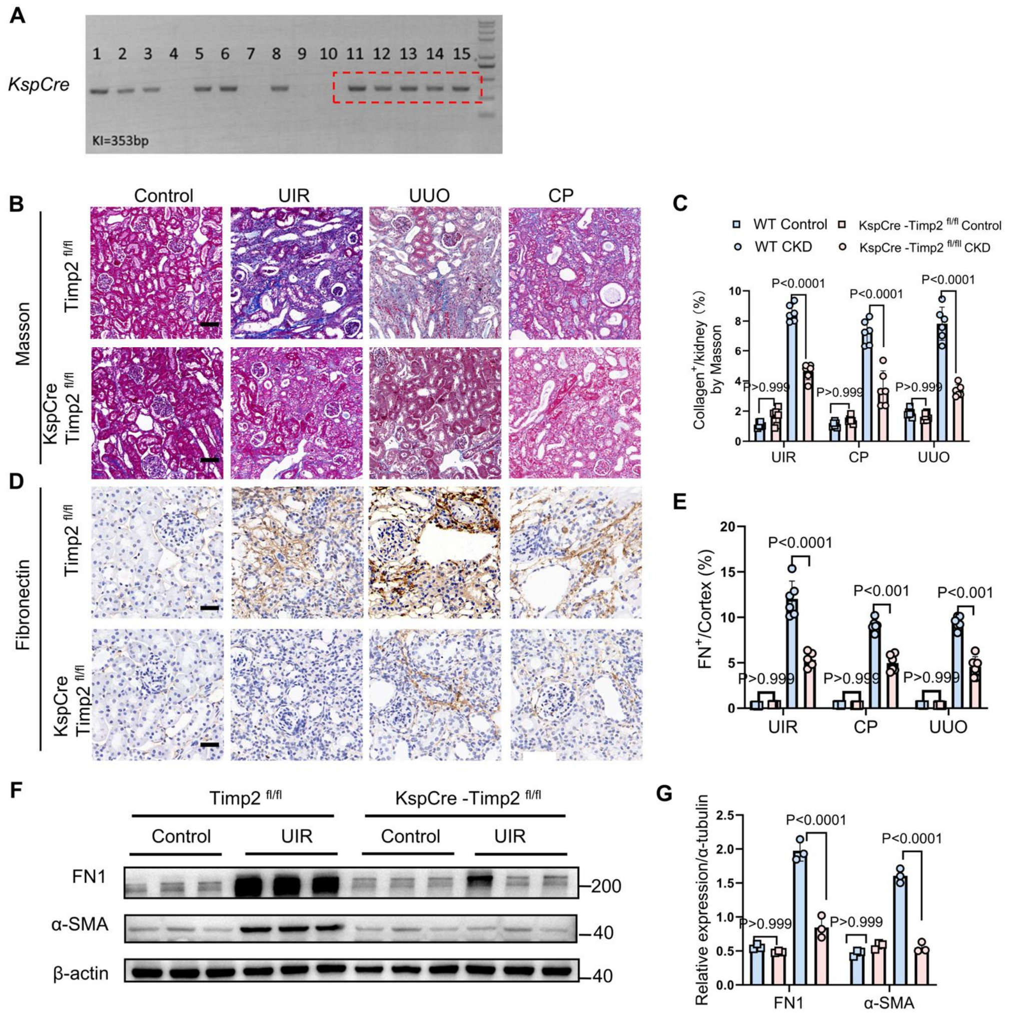

Supplementary Figure 3 Deletion of TIMP2 Reduces Fibrosis and Renal Injury in CKD Models

A: Agarose gel electrophoresis depicting bands for Ksp-Cre. **B**: Masson's trichrome staining to evaluate collagen fiber deposition in Timp2 $^{fl/fl}$ and Timp2 knockout mice in control Sham, UIR, UUO and CP groups. The scale bar is 50 µm. **C**: Quantification of collagen fiber deposition in kidney by Masson (n=6, data are mean ± s.d, two-way ANOVA test). **D:** Immunohistochemical staining showing the expression of fibronectin (FN) in WT and Timp2 knockout mice across the Sham, UIR, CP, and UUO groups. The scale bar is 50 µm. **E**: Quantitative of FN expression between groups (n=6, data are mean ± s.d, two-way ANOVA test). **F**: Western blot analysis evaluates the expression levels of FN and alpha-smooth muscle actin (α-SMA) in Timp2 $^{fl/fl}$ and Timp2 knockout mice in both Sham and UIR groups. The scale bar is 50 µm. **G:** Quantitative analysis of of FN and α-SMA protein expression levels by Western blot at 14 days post-UIR (n=6, data are mean ± s.d, two-way ANOVA test). Statistical

significance compared to the Sham group is indicated by * for $p < 0.05$, ** for $p < 0.01$, and *** for $p < 0.001$.

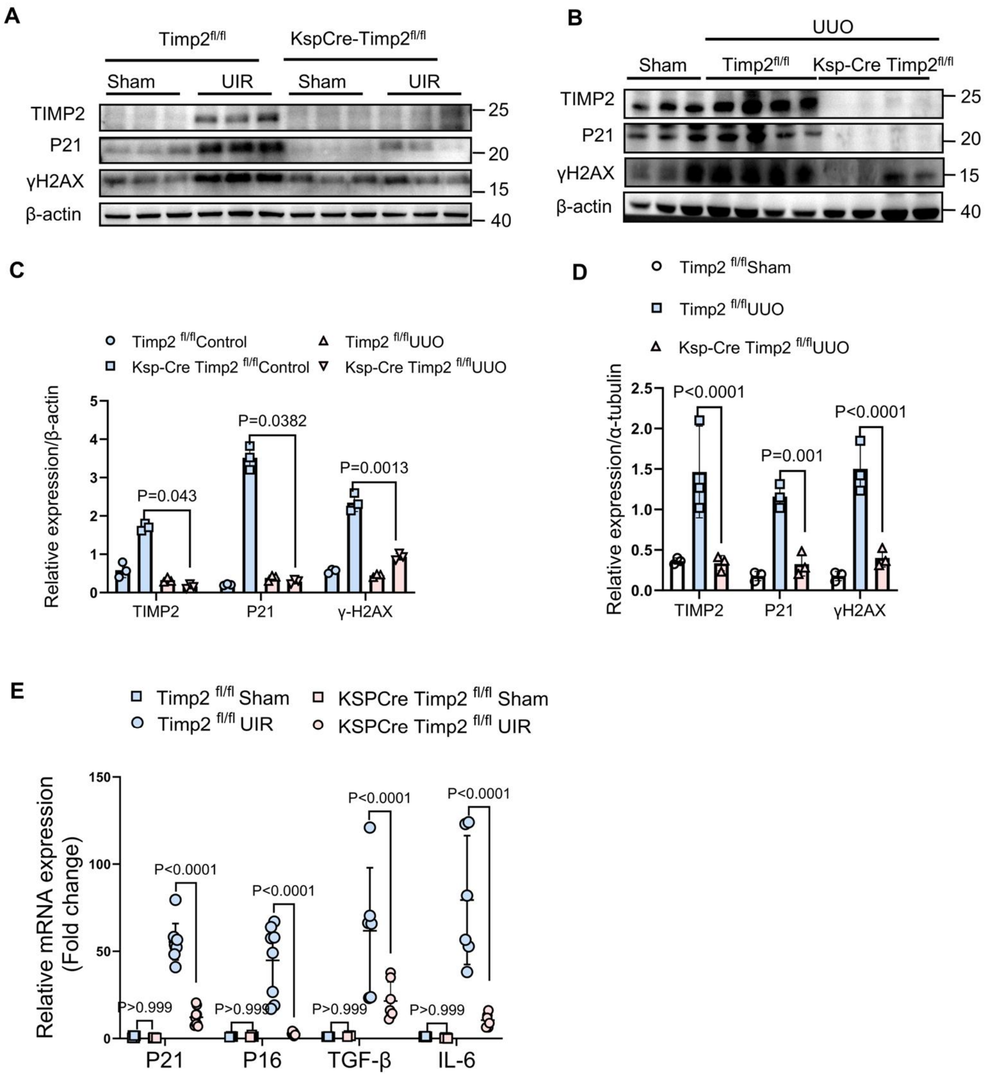

Supplementary Figure 4 TIMP2 Deletion Reduces the Expression of Cellular Senescence Markers

A-B: Western blot analysis showing the expression and quantitative statistics of TIMP2, senescence marker P21, and DNA damage marker γ-H2AX in WT and *KspCre- Timp2* $^{fl/fl}$ mice following UIR and UUO. **C-D:** Quantitative analysis of Western blot (n=6, data are mean ± s.d, two-way ANOVA test). **E:** Quantitative PCR analysis of the expression of senescence-associated genes p21, p16, Tgf-β, and Il-6 in the kidneys *Timp2*$^{fl/fl}$ and Timp2 knockout (*KspCre-Timp2*$^{fl/fl)}$ mice post-UIR (n=6, data are mean ± s.d, two-way ANOVA test).

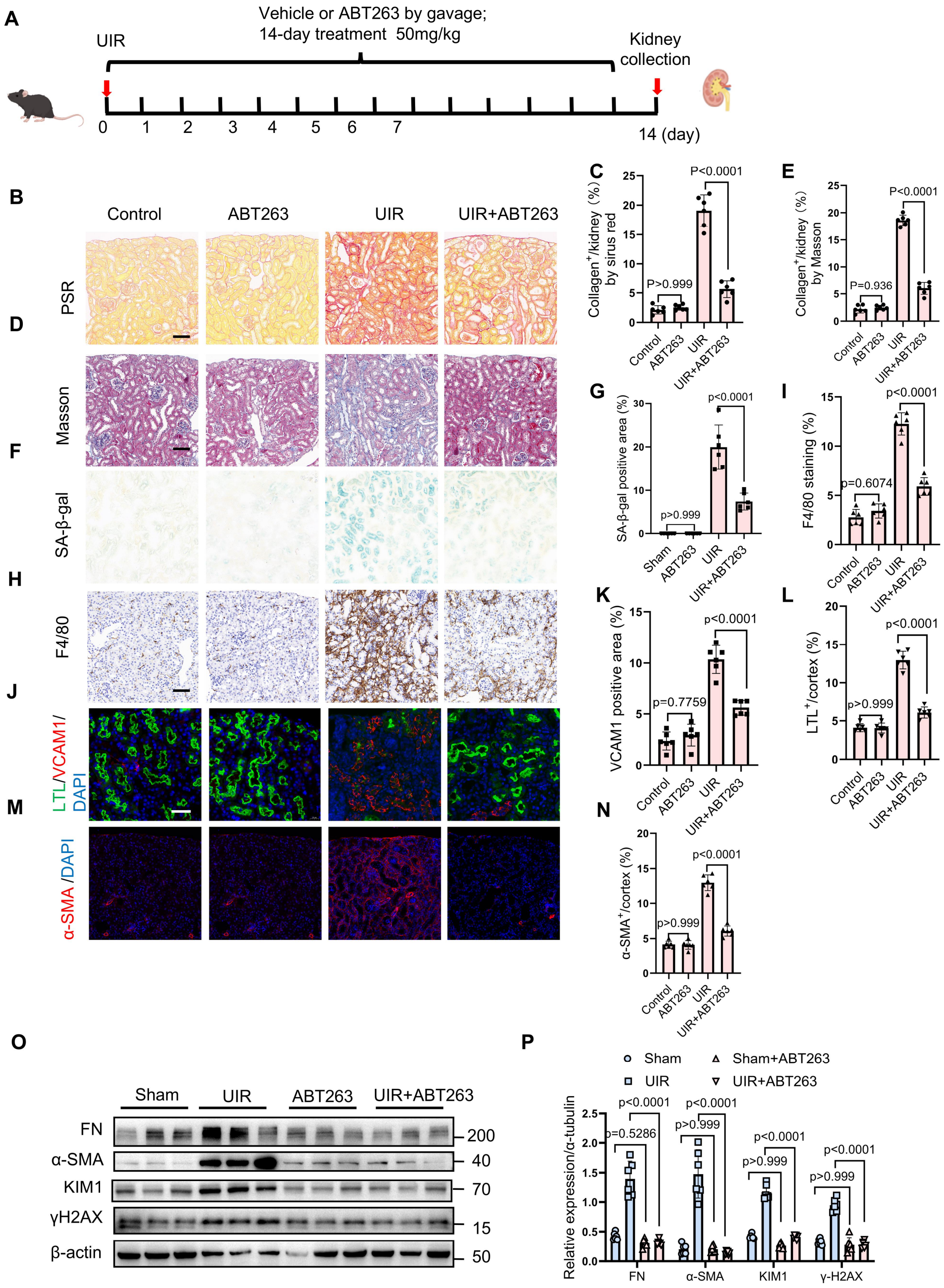

Supplementary Figure 5 Clearance of senescent cells suppresses maladaptive repair and alleviates renal interstitial fibrosis.

A: Experimental flowchart depicting the administration of ABT-263 (50 mg/kg) via oral gavage to mice subjected to UIR. ABT-263 was administered on the day of UIR

and daily thereafter until the day before tissue collection. **B:** PSR staining was used to quantify the deposition of collagen fibers in the kidneys of UIR mice and provide statistical analysis. ABT-263 significantly reduced the collagen fiber deposition induced by UIR. Scale bar is 50 μm. **C:** Quantification of collagen fiber deposition in kidney by PSR (n=6, data are mean ± s.d, one-way ANOVA test). **D:** Masson's trichrome staining assessed the degree of renal fibrosis in mice treated with ABT-263 and provided quantitative statistics. **E:** Quantification of collagen fiber deposition in kidney by Masson (n=6, data are mean ± s.d, one-way ANOVA test). **F:** SA-β-galactosidase staining showing differences in staining and quantitative analysis across treatment groups. **G:** Quantification of cell senescence in kidney by SA-β-galactosidase staining (n=6, data are mean ± s.d, one-way ANOVA test). **H:** F4/80 immunohistochemical staining marked the inflammatory macrophage infiltration in the kidneys of UIR mice and provided quantitative statistics. Scale bar is 50 μm. Each group consisted of n=6. **I:** Quantification of Inflammatory cell infiltration in kidney by F4/80 (n=6, data are mean ± s.d, one-way ANOVA test). **J:** Immunofluorescence staining of VCAM1 (red) to mark cellular repair processes. Scale bar is 50μm. Each group consisted of 6 samples (n = 6). **K:** Quantitative analysis of VCAM^{+} area/cortex (%) (n=6, data are mean ± s.d, one-way ANOVA test). **L:** Quantitative analysis of LTL^{+} area/cortex (%) (n=6, data are mean ± s.d, one-way ANOVA test). **M:** Immunofluorescence staining of α-SMA (n=6). The scale bar is 50 μm. **N**: Quantitative analysis of α-SMA^{+} area/cortex (%) (n=6, data are mean ± s.d, one-way ANOVA test). **O:** Western blot showing the expression levels and quantitative statistics of FN, α-SMA KIM1 and γH2AX in mice kidney after UIR with or without ABT163. **P:** Quantitative analysis of of FN, α-SMA KIM1 and γH2AX protein expression levels by Western blot at 14 days post-UIR (n=6, data are mean ± s.d, one-way ANOVA test).

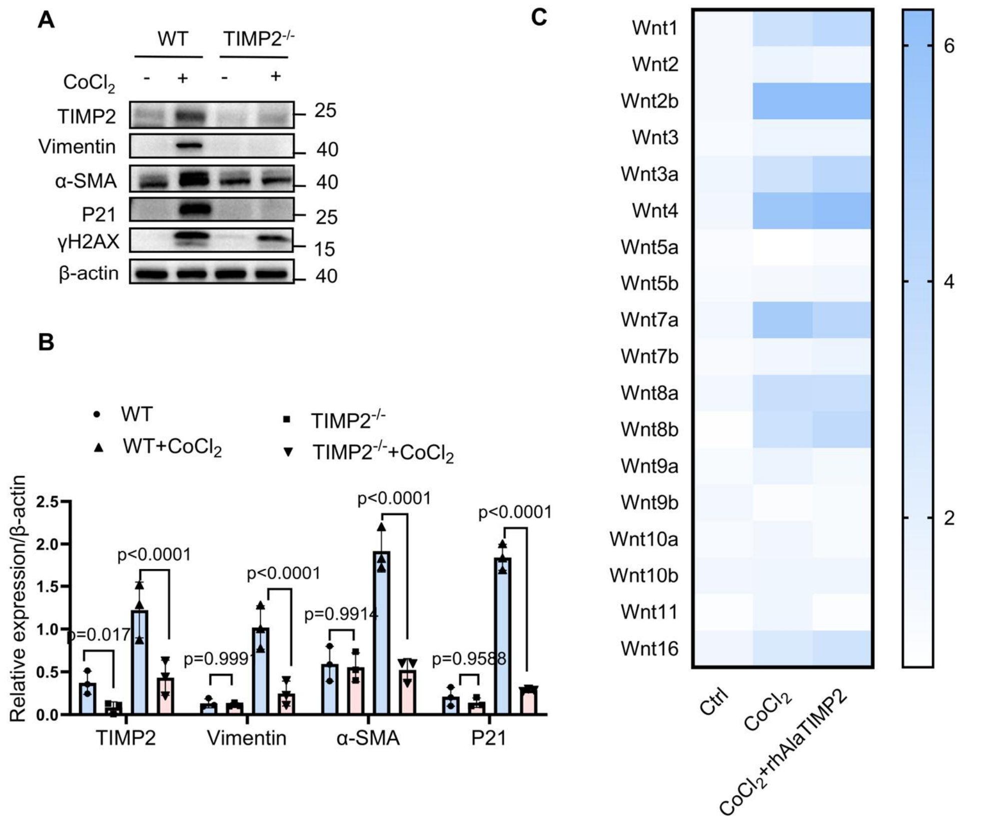

Supplementary Figure 6 Ablation of TIMP2 reduces senescence without altering Wnt ligand expression.

A: Western blot showing the expression levels and quantitative statistics of TIMP2, Vimentin, α-SMA, P21 and γH2AX in PRTCs with or without $CoCl_2$. **B:** Quantitative analysis of of FN, α-SMA KIM1 and γH2AX protein expression levels by Western blot in PRTCs with or without $CoCl_{2.}$ n=3. Data are mean ± s.d, two-way ANOVA test. **C:** Quantitative RT-PCR analysis of the mRNA expression levels of all 19 Wnt ligands in PRTCs under different conditions, comparing WT group, CoCl2- treated group, and TIMP2-KO+ CoCl2 treated group (n=3 Data are mean ± s.d, one-way ANOVA test). Experiments were repeated in three biological replicates using primary cells isolated from different mice. Experiments were repeated in three biological replicates using primary cells isolated from different mice.

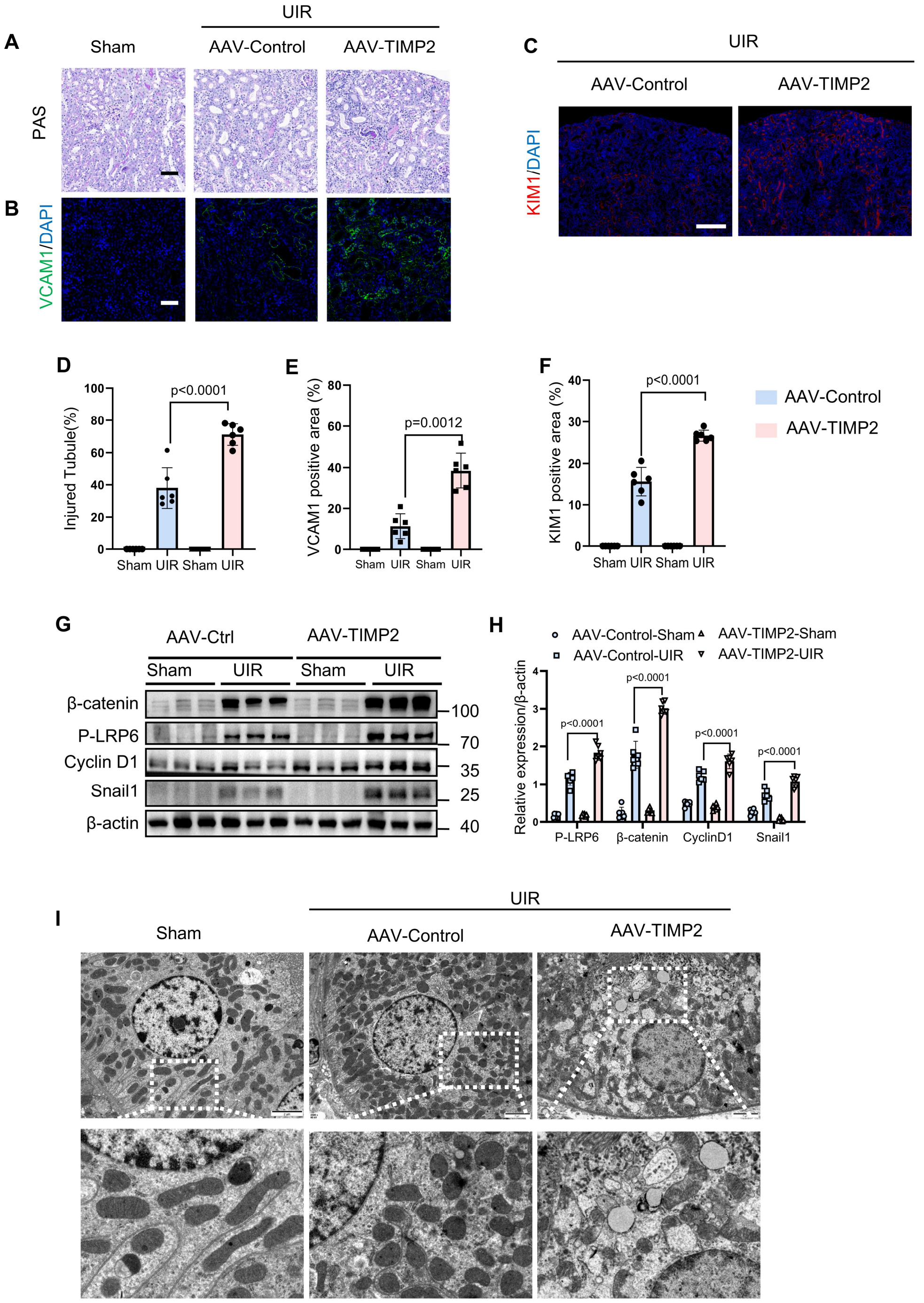

Supplementary Figure 7 TIMP2 overexpression aggravates renal injury and maladaptive repair and hyperactivation of the β-catenin pathway

A: PAS staining of kidney tissues after UIR. The scale bar is 50 μm. **B:** Immunofluorescence staining showing the expression of the VCAM1 in AAV-Control

and AAV-TIMP2 mice within UIR model. The scale bar is 50 μm. **C:** Immunofluorescence staining showing the expression of the KIM1 in AAV-Control and AAV-TIMP2 mice within UIR model. The scale bar is 50 μm. **D:** Quantification of kidney injury by PAS (n=6). **E:** Quantitative analysis of VCAM1+ area/cortex (%). n=6. **F:** Quantitative analysis of KIM1+ area/cortex (%). n=6. **G:** Western blot showing the expression levels and quantitative statistics of β-catenin, P-LRP6, Cyclin D1 and Snail1 in mice kidney after UIR with or without ABT163. **H:** Quantitative analysis of β-catenin, P-LRP6, Cyclin D1 and Snail1 protein expression levels by Western blot at 14 days post-UIR (n=3). **I:** Representative transmission electron microscopy (TEM) images of tissue sections. Red arrows: Indicating mitochondrial damage. Red dashed boxes: Areas shown in magnified insets. Data are mean ± s.d, two-way ANOVA test.
